# Associations between positive end-expiratory pressure and outcome of patients without ARDS at onset of ventilation: a systematic review and meta-analysis of randomized controlled trials

**DOI:** 10.1186/s13613-016-0208-7

**Published:** 2016-11-03

**Authors:** Ary Serpa Neto, Roberto Rabello Filho, Thomas Cherpanath, Rogier Determann, Dave A. Dongelmans, Frederique Paulus, Pieter Roel Tuinman, Paolo Pelosi, Marcelo Gama de Abreu, Marcus J. Schultz, For the PROVE Network Investigators

**Affiliations:** 1 Department of Critical Care Medicine, Hospital Israelita Albert Einstein, São Paulo, Brazil; 2 Department of Intensive Care, Academic Medical Center, University of Amsterdam, Amsterdam, The Netherlands; 3 Department of Critical Care, Westfriesgasthuis, Hoorn, The Netherlands; 4 National Intensive Care Evaluation, Amsterdam, The Netherlands; 5 Department of Intensive Care & REVIVE Research VUmc Intensive Care, Free University Medical Center, Amsterdam, The Netherlands; 6 Department of Surgical Sciences and Integrated Diagnostics, IRCCS AOU San Martino IST, University of Genoa, Genoa, Italy; 7 Department of Anesthesiology and Intensive Care Medicine, Pulmonary Engineering Groups, University Hospital Carl Gustav Carus, Technische Universität Dresden, Dresden, Germany; 8 Laboratory of Experimental Intensive Care and Anesthesiology, Academic Medical Center, University of Amsterdam, Amsterdam, The Netherlands

**Keywords:** Mechanical ventilation, Positive end-expiratory pressure, Intensive care unit, Acute respiratory distress syndrome, Atelectasis, Hyperinflation, Meta-analysis

## Abstract

**Background:**

The aim of this investigation was to compare ventilation at different levels of positive end-expiratory pressure (PEEP) with regard to clinical important outcomes of intensive care unit (ICU) patients without acute respiratory distress syndrome (ARDS) at onset of ventilation.

**Methods:**

Meta-analysis of randomized controlled trials (RCTs) comparing a lower level of PEEP with a higher level of PEEP was performed. The primary outcome was in-hospital mortality.

**Results:**

Twenty-one RCTs (1393 patients) were eligible. PEEP ranged from 0 to 10 cmH_2_O and from 5 to 30 cmH_2_O in the lower PEEP and the higher PEEP arms of included RCTs, respectively. In-hospital mortality was not different between the two PEEP arms in seven RCTs (risk ratio [RR] 0.87; 95% confidence interval [CI] 0.62–1.21; *I* ^2^ = 26%, low quality of evidence [QoE]), as was duration of mechanical ventilation in three RCTs (standardized mean difference [SMD] 0.68; 95% CI −0.24 to 1.61; *I* ^2^ = 82%, very low QoE). PaO_2_/FiO_2_ was higher in the higher PEEP arms in five RCTs (SMD 0.72; 95% CI 0.10–1.35; *I* ^2^ = 86%, very low QoE). Development of ARDS and the occurrence of hypoxemia (2 RCTs) were lower in the higher PEEP arms in four RCTs and two RCTs, respectively (RR 0.43; 95% CI 0.21–0.91; *I* ^2^ = 56%, low QoE; RR 0.42; 95%–CI 0.19–0.92; *I* ^2^ = 19%, low QoE). There was no association between the level of PEEP and any hemodynamic parameter (four RCTs).

**Conclusion:**

Ventilation with higher levels of PEEP in ICU patients without ARDS at onset of ventilation was not associated with lower in-hospital mortality or shorter duration of ventilation, but with a lower incidence of ARDS and hypoxemia, as well as higher PaO_2_/FiO_2_. These findings should be interpreted with caution, as heterogeneity was moderate to high, the QoE was low to very low, and the available studies prevented us from addressing the effects of moderate levels of PEEP.

**Electronic supplementary material:**

The online version of this article (doi:10.1186/s13613-016-0208-7) contains supplementary material, which is available to authorized users.

## Background

Mechanical ventilation can be a lifesaving strategy in critically ill patients, but there is unequivocal evidence that it can aggravate, or even initiate lung injury [1]. Indeed, invasive positive pressure ventilation and sedation may contribute to development of atelectasis [2, 3], increasing the risk of repetitive opening and closing of atelectatic lung tissue, so-called atelectrauma [1]. Results from preclinical studies using animals [4, 5] and studies in humans [6, 7] support the use of positive end-expiratory pressure (PEEP), to prevent or at least minimize atelectrauma. PEEP, however, can also lead to lung injury due to overdistension [8, 9], so-called volutrauma [1].

Atelectasis is more extensive in patients with the acute respiratory distress syndrome (ARDS) than in patients without lung injury and is more frequently seen with mandatory than spontaneous forms of ventilation [10, 11]. In patients with ARDS, therefore, the balance between prevention of atelectrauma and induction of overdistension could result in a net beneficial effect. In patients without ARDS, who more frequently receive spontaneous forms of ventilation, the balance between benefit and harm could go in the other direction since benefit of PEEP with less atelectasis is reduced. One meta-analysis of three large randomized controlled trials (RCTs) comparing higher to lower levels of PEEP in patients with ARDS showed benefit of higher levels of PEEP, albeit only in patients with more severe form of ARDS [12]. Sufficiently large RCTs comparing higher to lower levels of PEEP in patients without ARDS are lacking.

Besides increasing lung aeration, PEEP has also extrapulmonary effects. PEEP affects the loading conditions of the heart [13], as every increase in intrathoracic pressure reduces the preload of the heart and might increase as well as decrease the afterload of the right ventricle depending on whether lung tissue is recruited [13]. The effects of PEEP on cardiac performance could also differ between patients with ARDS and patients without ARDS, as PEEP could reduce right ventricle afterload by preventing or minimizing atelectasis in ARDS patients, while only raising intrathoracic pressure in patients without ARDS [13]. Furthermore, the effects of PEEP on the systemic circulation depend not only on how much lung tissue is recruited but also on lung volume, since if the lung volume is below the functional residual capacity at end expiration, an increase in the level of PEEP likely increases the cardiac output [14].

We set out to address the potential role of PEEP in ventilation of critically ill patients without ARDS at onset of ventilation. Therefore, we conducted a meta-analysis of RCTs comparing ventilation with different levels of PEEP in patients without ARDS. In the literature of ventilation often the terms ‘low’ and ‘lower,’ and ‘high’ and ‘higher’ are used. In this systematic review and meta-analysis, ‘lower’ and ‘higher’ were used for the comparisons of the level of PEEP in the arms compared within each RCT. We hypothesized that ventilation with higher levels of PEEP is associated with improved survival and shorter duration of ventilation.

## Methods

### Search strategy

Studies were identified through an electronic search of PubMed (1966 till July 2016), CENTRAL (the Cochrane Library till July 2016), Clinicaltrials.gov (till July 2016), ICTRP (International Clinical Trials Registry Platform till July 2016), Web of Science (till July 2016) and CINAHL (till July 2016) by two blinded investigators. A search strategy incorporating keywords as well as utilizing Medical Subject Headings was used: (‘PEEP’ OR ‘positive end-expiratory pressure’ OR ‘positive end-expiratory pressure’ OR ‘positive end-expiratory pressure’) AND (‘randomized’ OR ‘RCT’). All articles returned for this query were scanned for relevancy by title and abstract. For potentially relevant articles, the full text was obtained for review; of these articles, as well as related reviews and meta-analyses, all references were inspected and potentially relevant titles were hand searched. No further limitations were set on the query. We also contacted leaders and experts in the field of ventilation of critically ill patients and asked them whether they were aware of recently finished or planned RCTs of ventilation with different levels of PEEP.

### Selection of studies

The following inclusion criteria were used: (1) RCTs of ventilation; (2) in adult patients without ARDS at onset in an ICU setting; (3) comparing different levels of PEEP in the randomization arms; (4) at a similar tidal volume. Observational and retrospective studies, RCTs that (also) concerned patients with ARDS, RCTs comparing strategy bundles (e.g., a low tidal volume plus higher levels of PEEP vs. high tidal volume plus lower levels of PEEP), RCTs in another setting than the ICU and RCTs comparing different levels of PEEP within one single patient were excluded.

### Data extraction and quality assessment of studies

Two investigators (ASN and RRF) extracted the data into a database developed for this particular dataset. Wherever they disagreed on data extraction, this was settled by discussion. The Cochrane Risk of Bias Tool was used to assess the quality of the studies.

### Definition of endpoints

The primary endpoint was in-hospital mortality at longest follow-up. Follow-up periods of mortality were highly variable and depended on the reported data in the retrieved articles. Secondary endpoints were: (1) 28-day mortality (proportion); (2) duration of mechanical ventilation (in days and for all patients); (3) development of pulmonary complications, including ARDS, pneumonia, atelectasis on chest radiographs or barotrauma (proportion); (4) incidence of hypoxemia (proportion) and the lowest PaO_2_/FiO_2_ (in mmHg); and (5) incidence of hypotension (proportion) and the lowest arterial blood pressure (in mmHg). The definition of each endpoint in the studies is shown in Additional file 1: Table S1.

### Statistical analysis

For the meta-analysis, we considered all the manuscripts included in the systematic review. All patients were analyzed in the study group to which they were randomized in the original study, i.e., the lower or higher PEEP arms (intention-to-treat principle). For dichotomous data, we calculated a pooled estimate of risk ratio (RR) in the individual studies using a random-effects model according to Mantel and Haenszel and graphically represented these results using forest plot graphs. We expressed pooled continuous effect measures as the standardized mean difference (SMD) with 95% confidence intervals (95% CI). The homogeneity assumption was measured by the *I* ^2^, which describes the percentage of total variation across studies that is due to heterogeneity rather than chance. *I* ^2^ was calculated from basic results obtained from a typical meta-analysis as *I* ^2^ = 100% × (*Q* − *df*)/*Q*, where *Q* is the Cochran’s heterogeneity statistic. A value of 0% indicates no observed heterogeneity, and larger values indicate increasing heterogeneity. Publication bias was addressed visually using a funnel plot. The GRADE approach was used to test the overall quality of evidence (QoE) [15].

Subgroup analyses were carried out by recalculating pooled RR estimates for different subgroups of studies for specific reasons as follows: (1) medical versus surgical patients, since the duration of ventilation as well as outcome in these two groups of patients is very different; (2) use of PEEP versus 0 cmH_2_O of PEEP (frequently called zero end-expiratory pressure, ZEEP), since the effects of using ZEEP could be very different from using a lower level of PEEP; (3) use of PEEP ≥ 10 versus PEEP < 10 cm H_2_O, since this level is the most accepted higher level of PEEP; and (4) RCTs published up to 2000 versus RCTs published after 2000, since several RCTs published at the beginning of this century clearly demonstrated that ventilation-induced lung injury is a true, but foremost preventable entity—this was by then demonstrated for tidal volume reduction, but it could very well have resulted in more changes in ventilation practice than changes in the tidal volume size used in ICU patients [1]. These analyses were performed to test whether the overall results were affected by a change in the meta-analysis selection criteria. A meta-regression was performed using tidal volume as covariate to check whether there was interaction between outcomes, levels of PEEP and the size of tidal volumes used.

Parametric variables were presented as the mean ± standard deviation (SD), and nonparametric variables were presented as the median (interquartile range). All analyses were performed with Review Manager version 5.1.1, SPSS version 20 (IBM SPSS Statistics for Windows, version 20.0. Armonk, NY: IBM Corporation) or R version 2.12.0 (R Foundation for Statistical Computing, Vienna, Austria). For all analyses, two-sided *p* values <0.05 were considered significant.

## Results

**Fig. 1:**
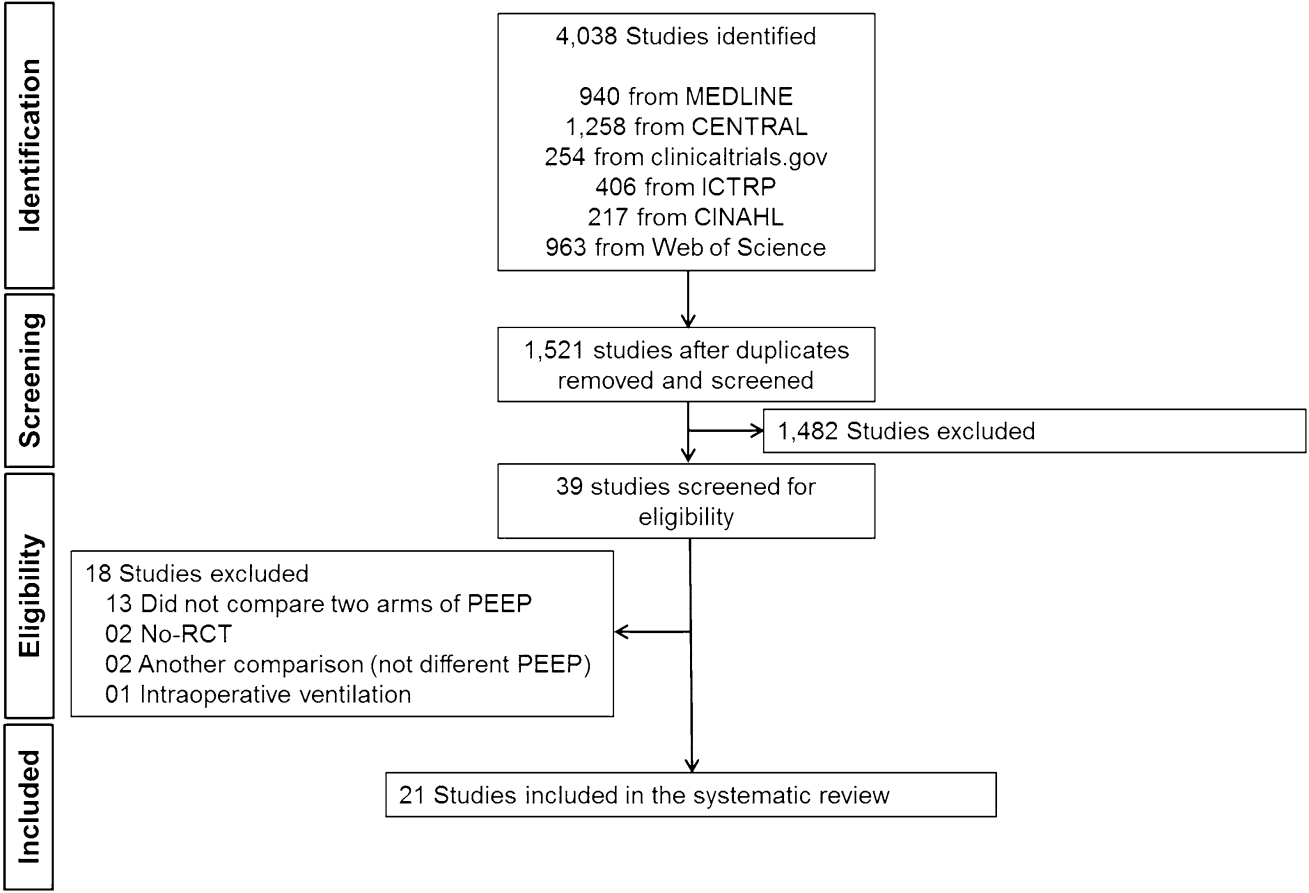
Flowchart of the systematic review

The initial search yielded 4038 articles (940 from MEDLINE, 1258 from CENTRAL, 254 from clinicaltrials.gov, 406 from ICTRP, 217 from CINAHL and 963 from Web of Science) (Fig. 1). Leaders and experts in the field of ventilation of critically ill patients did not report ongoing or recently finished RCTs. After removing duplicates, we evaluated the abstracts of 1521 articles, of which 1482 articles were excluded because they did not meet the inclusion criteria of this systematic review. Subsequently, we read the full text of each of the remaining 39 articles. Eighteen articles were excluded for the following reasons: (1) did not compare two or more PEEP strategies (*n* = 13); (2) no RCT (*n* = 2); (3) the comparisons included more than only a difference in PEEP (*n* = 2); and (4) ventilation outside an ICU setting (*n* = 1). Finally, 21 RCTs (1393 participants) were included in the meta-analysis [16–36].

Table 1 and Additional file 1: Tables S2 and S3 summarize the characteristics of the RCTs according to the type of patient analyzed. In most of the RCTs, ZEEP was used in the lower PEEP arm (61.9%); notably, these RCTs were published over 20 years ago (Additional file 1: Table S3). The size of tidal volumes was always similar in the randomization arms within each RCT [9.7 ± 2.7 (range 6–15) ml/kg], while the level of PEEP was 2.0 ± 2.8 (range 0–10) cmH_2_O and 9.7 ± 4.0 (range 5–30) cmH_2_O in the lower and the higher PEEP arms of the included RCTs, respectively (Additional file 1: Table S3). In 16 RCTs, the level of PEEP was set arbitrarily (Additional file 1: Table S2) and in five RCTs PEEP was titrated, e.g., by using PaO_2_ or a pressure–volume curve (Additional file 1: Table S2).

**Table 1:** Characteristics of included studies

Study	Year	Type of patients	*N*	High PEEP group					Low PEEP group					Main findings
				*N*	PEEP	*V*_T_	RM	DMV	*N*	PEEP	*V*_T_	RM	DMV	
*Surgical ICU patients*														
Lago Borges et al.	2014	Post-CG	136^b^	44	10	08	No	0.47	45	05	08	No	0.47	Shorter duration of ventilation with higher PEEP (considering only patients extubated within 12 h after ICU admission)
Lago Borges et al.	2013	Post-CG	136^b^	44	10	08	No	0.47	45	05	08	No	0.47	Higher compliance and less hypoxemia (P/F ratio < 300) with higher PEEP
Celebi et al.	2007	Post-CG	60^a^	20	10	07	Yes	0.25	20	05	07	No	0.25	Increase in P/F ratio (in the first 4 h but not after extubation) and less atelectasis with higher PEEP
Holland et al.	2007	Post-CG	28	14	10	6–8	No	0.08	14	05	6–8	No	0.08	No differences regarding cardiac index and filling pressures, liver function and gastric mucosal perfusion
Dyhr et al.	2002	Post-CG	16	08	15	06	Yes	NA	08	00	06	Yes	NA	Increase in P/F ratio and EELV (in the first 3 h) and less atelectasis with higher PEEP
Michalopoulos et al.	1996	Post-CG	67^d^	21	10	NA	No	NA	22	00	NA	No	NA	No differences regarding P/F ratio, duration of ventilation, and atelectasis
Carroll et al.	1988	Postsurgery P/F < 200	50	22	15	12	Yes	15	28	04	12	Yes	02	More hypotension, barotrauma, and death and higher duration of ventilation with higher PEEP
Marvel et al.	1986	Post-CG	44^c^	12	10	12	No	0.39	15	05	12	No	0.46	Lower alveolar–arterial oxygen tension gradient with higher PEEP (similar after extubation). No differences regarding atelectasis, and hospital length of stay
Murphy et al.	1983	Post-CG	139	NA	10	NA	No	0.33	NA	00	NA	No	0.33	No differences in blood loss independent of coagulation profile
Zurick et al.	1982	Post-CG	83	41	10	NA	No	NA	42	00	NA	No	NA	No differences regarding the amount of blood loss, need for re-exploration or blood requirement
Good et al.	1979	Post-CG	24	10	06	11	No	0.62	14	00	11	No	0.62	No differences regarding atelectasis, P/F ratio and arterial–alveolar ratio
Schmidt et al.	1976	Post-surgery risk of ARDS	112	56	08	12–15	No	NA	56	00	12–15	Yes	NA	Higher PaO_2_, alveolar–arterial oxygen tension, and lower incidence of ARDS and other pulmonary complications with higher PEEP
*Medical*														
Ma et al.	2014	NPE	120	60	11–30	6–8	No	NA	60	3–10	6–8	No	NA	Lower 28-day mortality, EVLWi, PVPi, blood pressure and higher P/F ratio with high PEEP
Lesur et al.	2010	ARF	63	30	05	08	No	9.2	33	00	07	No	9.2	No differences regarding hypotension, duration of ventilation and mortality (PEEP used only during 90 min)
Manzano et al.	2008	Clinical P/F > 250	127	64	5–8	08	No	4.5	63	00	08	No	5.6	No differences regarding hospital mortality, ARDS, atelectasis or barotrauma. Lower incidence of VAP and hypoxemia with high PEEP
Vigil et al.	1996	Trauma	44	23	05	12	No	3.2	21	00	12	No	3.6	No differences regarding intrapulmonary shunt, dead space and P/F ratio
Cujec et al.	1993	ARF	46	NA	10	NA	No	NA	NA	00	NA	No	NA	Reduction in alveolar–arterial oxygen difference and shunt fraction with higher PEEP
Nelson et al.	1987	P/F < 250	38	20	15	NA	No	5.3	18	08	NA	No	3.4	No differences in duration of ventilation, ICU and hospital length of stay, barotrauma, and mortality
Pepe et al.	1984	Risk of ARDS	92	44	08	12	No	3.0	48	00	12	No	3.0	No differences regarding ARDS, barotrauma, atelectasis, mortality, duration of ventilation, ICU length of stay
Weigelt et al.	1979	Risk of ARDS	79	45	05	15	No	5.0	34	00	15	No	8.0	Lower incidence of ARDS, and pulmonary mortality and higher incidence of pulmonary dysfunction with higher PEEP
Feeley et al.	1975	ARF	25	12	05	10	No	NA	13	00	10	No	NA	Improve in vital capacity and the maximum inspiratory force and less increase in intra-pulmonary shunt with higher PEEP

PEEP: positive end-expiratory pressure (in cmH_2_O); V_T_: tidal volume (in ml/kg); RM: recruitment maneuvers; DMV: duration of mechanical ventilation (in days); P/F: PaO_2_/FiO_2_; CG: cardiac surgery; NA: not available; EELV: end-expiratory lung volume; ARF: acute respiratory failure; NPE: neurologic pulmonary edema; EVLWi: extravascular lung water index; PVPi: pulmonary vascular permeability index; ARDS: acute respiratory distress syndrome; VAP: ventilator-associated pneumonia

^a^One group of 20 patients not included (use of CPAP only)

^b^One group of 47 patients not included (intermediate level of PEEP [8 cmH_2_O])

^c^ One group of 17 patients not included (exhaled to ambient pressure)

^d^ One group of 24 patients not included (intermediate level of PEEP [5 cmH_2_O])

The quality of the eligible RCTs is shown in Additional file 1: Figures S1 and S2. Most of the RCTs had high or unclear risk of bias. The high risk of bias was in the domains related to the blindness of participants, personnel and outcome assessors. RCTs were published between 1975 and 2014 and included surgical ICU patients (cardiac surgery: *n* = 10; non-cardiac surgery: *n* = 2) or medical ICU patients (*n* = 10). Duration of ventilation ranged from 2 to 360 h and from 3 to 9 days in surgical and medical ICU patients, respectively; duration of ventilation was not reported for seven RCTs.

### Primary endpoint

**Fig. 2:**
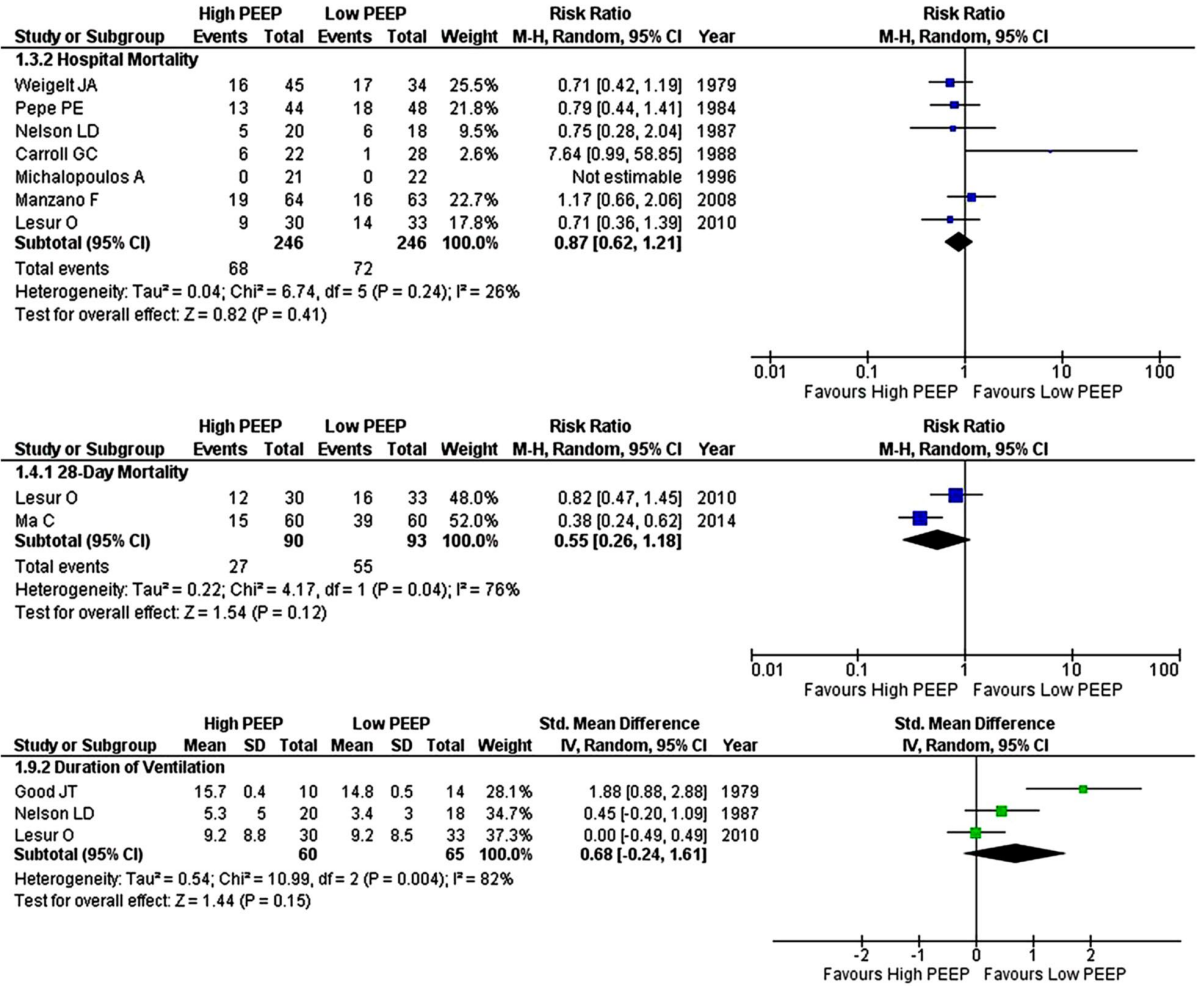
Forest plot of clinical outcomes: in-hospital mortality, 28-day mortality and duration of ventilation

Seven RCTs in the meta-analysis addressed in-hospital mortality. Sixty-eight out of 246 patients (27.6%) assigned to higher PEEP and 72 out of 246 patients (29.3) assigned to lower PEEP died during hospital stay (RR 0.87; 95% CI 0.62–1.21) (Fig. 2). There was low heterogeneity (*I* ^2^ = 26%; *p* = 0.24). Heterogeneity in the analysis of hospital mortality was caused by two RCTs conducted in postsurgical patients [21, 22]. Indeed, by removing these two RCTs from the meta-analysis heterogeneity disappeared (*I* ^2^ = 0%) (Table 2).

**Table 2:** Summary of stratified analyses of pooled risk ratios

Stratified analysis	No. of trials	No. of patients^a^	Risk ratio (95% CI) SMD (95% CI)	*p* value	*I* ^2^ (*p* value)
*In-hospital mortality*					
Level of PEEP					
High PEEP versus ZEEP	5	404	0.83 (0.62–1.11)	0.20	0% (0.57)
Type of patient					
Medical patients	5	399	0.82 (0.62–1.09)	0.17	0% (0.73)
Level of PEEP					
≥10 Versus <10 cmH_2_O	3	131	2.04 (0.19–21.85)	0.56	77% (0.04)
*28-Day mortality*					
Level of PEEP					
High PEEP versus ZEEP	1	63	0.82 (0.47–1.45)	0.50	NA
Type of patient					
Medical patients	2	183	0.55 (0.26–1.18)	0.12	76% (0.04)
Level of PEEP					
≥10 versus <10 cmH_2_O	1	120	0.38 (0.24–0.62)	<0.001	NA
*Duration of ventilation*					
Level of PEEP					
High PEEP versus ZEEP	2	87	0.89 (−0.95–2.73)	0.34	91% (< 0.01)
Type of patient					
Medical patients	2	101	0.17 (−0.25–0.60)	0.43	13% (0.28)
Level of PEEP					
≥10 versus <10 cmH_2_O	1	38	0.45 (−0.20–1.09)	0.18	NA
*ARDS*					
Level of PEEP					
High PEEP versus ZEEP	4	410	0.43 (0.21–0.91)	0.03	56% (0.08)
Type of patient					
Medical patients	3	298	0.52 (0.27–1.02)	0.06	50% (0.13)
Level of PEEP					
≥10 versus <10 cmH_2_O	0	0	NA	NA	NA
*Pneumonia*					
Level of PEEP					
High PEEP versus ZEEP	3	331	0.58 (0.29–1.15)	0.12	58% (0.09)
Type of patient					
Medical patients	2	219	0.64 (0.24–1.66)	0.36	72% (0.06)
Level of PEEP					
≥10 versus <10 cmH_2_O	0	0	NA	NA	NA
*Atelectasis*					
Level of PEEP					
High PEEP versus ZEEP	3	331	0.74 (0.33–1.66)	0.46	74% (0.02)
Type of patient					
Medical patients	2	219	1.00 (0.54–1.84)	0.99	63% (0.10)
Level of PEEP					
≥10 versus <10 cmH_2_O	0	0	NA	NA	NA
*Barotrauma*					
Level of PEEP					
High PEEP versus ZEEP	4	374	0.60 (0.13–2.70)	0.50	52% (0.15)
Type of patient					
Medical patients	3	257	0.79 (0.28–2.27)	0.67	22% (0.28)
Level of PEEP					
≥10 versus <10 cmH_2_O	4	203	6.26 (0.78–50.47)	0.09	0% (0.46)
*PaO*_*2*_ */FiO*_*2*_ *ratio*					
Level of PEEP					
High PEEP versus ZEEP	2	143	0.63 (0.29–0.96)	< 0.01	0% (0.79)
Type of patient					
Medical patients	2	247	1.17 (0.06–2.28)	0.04	94% (< 0.01)
Level of PEEP					
≥10 versus <10 cmH_2_O	4	253	0.74 (−0.14–1.61)	0.10	89% (< 0.01)
*Hypoxemia*					
Level of PEEP					
High PEEP versus ZEEP	2	170	0.42 (0.19–0.92)	0.03	19% (0.27)
Type of patient					
Medical patients	1	127	0.35 (0.20–0.61)	< 0.01	NA
Level of PEEP					
≥10 versus <10 cmH_2_O	1	43	1.05 (0.16–6.77)	0.96	NA
*Blood pressure*					
Level of PEEP					
High PEEP versus ZEEP	NA	NA	NA	NA	NA
Type of patient					
Medical patients	1	120	−0.92 (−1.30 to –0.54)	< 0.01	NA
Level of PEEP					
≥10 versus <10 cmH_2_O	2	158	−0.21 (−1.68 to 1.26)	0.78	92% (< 0.01)
*Hypotension*					
Level of PEEP					
High PEEP versus ZEEP	1	63	1.10 (0.68–1.79)	0.70	NA
Type of patient					
Medical patients	1	63	1.10 (0.68–1.79)	0.70	NA
Level of PEEP					
≥10 versus <10 cmH_2_O	1	82	32.16 (2.04–507.16)	0.01	NA

*CI* confidence interval, *SMD* standardized mean difference, *ICU* intensive care unit, *PEEP* positive end-expiratory pressure, *ZEEP* zero positive end-expiratory pressure, *ARDS* acute respiratory distress syndrome, *NA* not applicable

^a^Considering the studies included in each subgroup analysis

Visual inspection of the funnel plot suggested a risk of publication bias (Additional file 1: Figure S3), though interpretation of the funnel plot was hampered due to the fact that the number of RCTs was low. Consequently, the power of this test was too low to distinguish chance from real asymmetry.

Based on the GRADE approach, the overall quality of evidence (QoE) was low (Additional file 1: Table S4). According to the meta-regression, there was no interaction between the size of tidal volumes and the level of PEEP, and in-hospital mortality (*p* = 0.431) (Additional file 1: Figure S3).

### Secondary endpoints

There were no differences in 28-day mortality (analyzed in two RCTs) and duration of ventilation between the PEEP arms (three RCTs) (Fig. 2). Heterogeneity in the analysis of duration of ventilation was partly caused by one RCT in postsurgical patients [17]. Removing this RCT lowered the heterogeneity (*I* ^2^ = 13%) (Table 2).

**Fig. 3:**
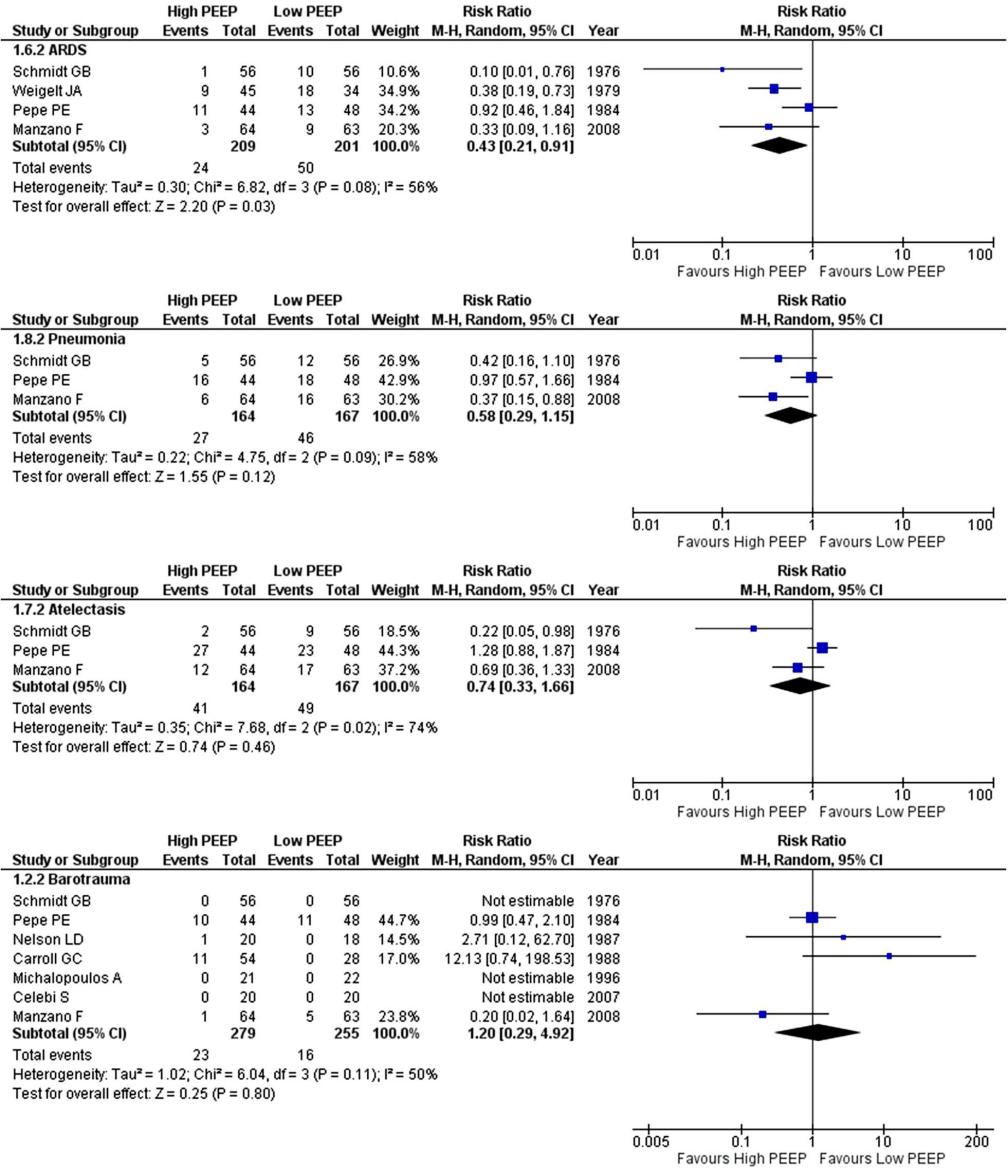
Forest plot of pulmonary complications: ARDS, pneumonia, atelectasis and barotrauma

**Fig. 4:**
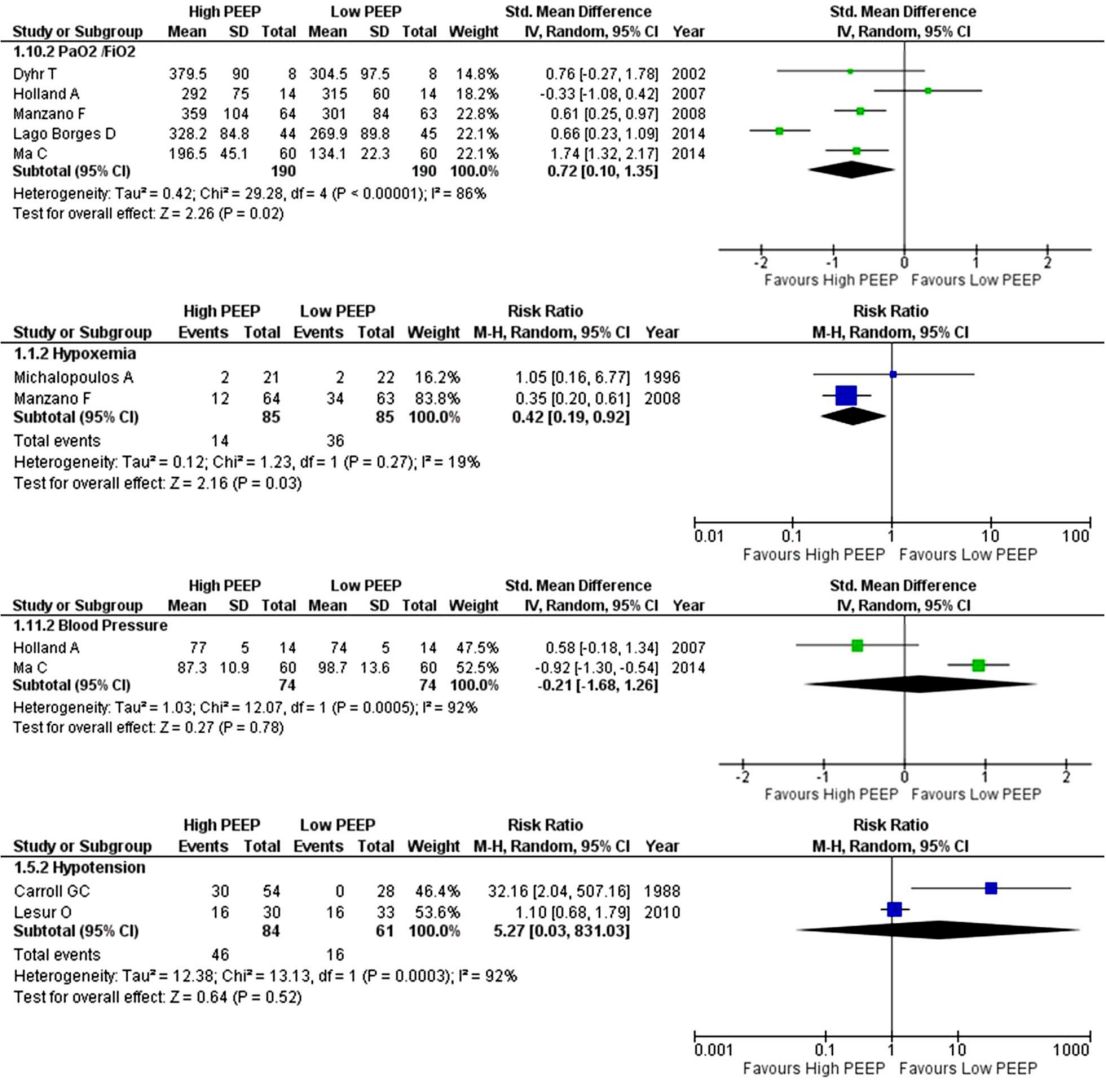
Forest plot of systemic effects: PaO_2_/FiO_2_, hypoxia, blood pressure and hypotension

The incidence of ARDS and hypoxemia was lower in the higher PEEP arms (three and two RCTs, respectively) (Figs. 3, 4). The PaO_2_/FiO_2_ during follow-up was higher in the higher PEEP arms (four RCTs) (Fig. 4). There were signs of moderate to high heterogeneity in these two analyses due to the inclusion of surgical ICU patients. Heterogeneity in the analysis of ARDS was totally caused by one RCT in patients at a very high risk of ARDS [30]. By removing this study, heterogeneity disappeared (*I* ^2^ = 0%). According to the meta-regression, there was no interaction between tidal volume size and the level of PEEP, and ARDS (*p* = 0.588) or PaO_2_/FiO_2_ (*p* = 0.824) (Additional file 1: Figure S3).

There were no differences in the incidence of pneumonia (three RCTs), atelectasis (three RCTs), barotrauma (seven RCTs), hypotension (two RCTs) and blood pressure levels (two RCTs) between the two groups (Figs. 3, 4). According to the meta-regression, there was no interaction between tidal volume size and the level of PEEP, pneumonia (*p* = 0.400), atelectasis (*p* = 0.879) or barotrauma (*p* = 0.188) (Additional file 1: Figure S3).

Visual inspection of the funnel plots suggested a risk of publication bias (Additional file 1: Figure S4), but again the power of this test was too low to distinguish chance from real asymmetry due to the fact that the numbers of RCTs were low. For all these analyses, based on the GRADE approach, the overall QoE was very low (Additional file 1: Table S4).

### Subgroup analyses

Subgroup analyses did not change the findings of the primary analysis (Table 2 and Additional file 1: Figures S5, 6, 7, 8, 9, 10, 11, 12 and 13) except for the association between the level of PEEP and the incidence of barotrauma, which was significant only in RCTs published before 2000 (*p* = 0.02) (Additional file 1: Figures S11, S12 and S13).

The analysis focusing on studies assessing PEEP levels ≥10 cmH2O in the high PEEP arm did not show different results from the overall analysis. Although there was a suggestion of reduction in 28-day mortality and increase in barotrauma with the use of PEEP levels ≥10 cmH2O, these results came from one single trial.

## Discussion

This systematic review and meta-analysis of RCTs in patients without ARDS did not find benefit from ventilation with higher levels of PEEP with regard to mortality, and duration of ventilation, neither in surgical ICU patients nor in medical ICU patients. Ventilation with higher levels of PEEP was associated with fewer occurrences of ARDS and less hypoxemia; however, the heterogeneity in these analyses was moderate to high and the QoE was low to very low. Subgroup analyses did not change the findings.

This is the first systematic review and meta-analysis investigating the association between ventilation with different levels of PEEP and clinical outcomes of ICU patients without ARDS. The included studies compared two different levels of PEEP using the same tidal volume in the two arms, thereby minimizing the risk of confounding effects of different tidal volume sizes. Indeed, as low tidal volume ventilation is associated with improved outcomes in patients without ARDS [37, 38], excluding RCTs comparing not only different levels of PEEP but also different tidal volume sizes increased the change that the associations, or absence of associations, were solely caused by the level of PEEP.

A subgroup analysis of medical ICU patients was performed because the duration of ventilation and the prognosis of this group of patients differ from patients’ receiving ventilation after elective surgery, and different outcomes would then be expected. The subgroup analysis showed no differences with the main analysis, but importantly there was no difference in occurrence of ARDS and blood pressure was lower in patients receiving higher PEEP compared to lower PEEP.

This systematic review and meta-analysis adds to our knowledge of the effects of PEEP in ICU patients without ARDS. First higher PEEP was associated with reduced incidence of ARDS and hypoxemia. This can possibly be explained by differences in the definitions of pulmonary complications like pneumonia and ARDS over the years; also, the present analysis showed moderate heterogeneity and a significant impact of PEEP was found only in older RCTs [16, 29]. One important shortcoming in the diagnosis of ARDS is that it is not standardized how to collect the PaO_2_/FiO_2_. Hypoxemia could simply disappear or become less worse by increasing the level of PEEP [39]. We remain uncertain whether this may have had an effect on the findings of the present analysis.

The RCTs performed so far were in general too small and mainly assessed outcomes that could suffer from bias, like development of pulmonary complications. Notably, standard ventilatory care has changed considerably over the last decades, as did the way in which pulmonary complications like pneumonia and ARDS were to be scored, mainly due to new definitions. In the present analysis, the use of higher levels of PEEP was associated with lower incidence of ARDS. However, the present analysis showed moderate heterogeneity and a significant impact of PEEP was found only in older RCTs [16, 29]. Thus, these ‘older’ RCTs had a higher incidence of ARDS than more recent RCTs, and it could very well be that what now is scored as atelectasis, then was scored as ARDS. This is also supported by the fact that mortality was not affected in the older RCTs, which one would have expected when more patients develop ARDS [16, 29, 30]. However, all these older trials were underpowered to address mortality as outcome.

Notably, the size tidal volume differed between RCTs, with higher tidal volumes in the older RCTs. This could have augmented the risk of secondary injury in patients without ARDS [37, 38], and PEEP may have had other effects when higher tidal volumes are used, although this remains completely speculative. Substantial evidence indicates that mechanical ventilation *per se* contributed to the development of ARDS, i.e., through ventilation-induced lung injury (VILI) [1]. The repetitive opening and closing of lung units during each respiratory cycle is one of the mechanisms responsible for VILI, and strategies of ventilation focusing on the use of recruitment maneuvers and application of higher PEEP can prevent this [40]. Indeed, clinical studies have shown that ventilation with higher levels of PEEP reduces the incidence of atelectasis [6, 41, 42] and improves respiratory system compliance mainly in patients receiving general anesthesia for surgery [43, 44]. Also, higher levels of PEEP could alleviated lung inhomogeneity, decreasing the impact of the stress raisers and the stress concentration due to inflation of well-aerated alveoli adjacent to collapsed or fluid-filled alveoli [45, 46].

Recently, the effects of PEEP gained increasing interest from anesthesiologists, who struggle with the same question of whether or not to apply PEEP during intraoperative ventilation in surgical patients. Three randomized controlled trials in surgical patients showed that the use of higher levels of PEEP with recruitment maneuvers combined with low tidal volumes was associated with better outcomes compared to conventional ventilation [47–49]. Thus, these RCTs studied the effect of a bundle of ventilator settings that are all expected to have an effect on pulmonary integrity. One more recent randomized controlled trial, however, showed no difference in the incidence of pulmonary complication when two different levels of PEEP were compared during low tidal volume ventilation [50]. Interestingly in this context is that a recent individual patient meta-analysis of intraoperative ventilation settings suggests that benefit mainly comes from tidal volume reductions, and not increases in PEEP, in patients undergoing mechanical ventilation for general anesthesia for surgery [51].

An important point is the difference between using an arbitrary higher level of PEEP or to titrate the PEEP according to your patient’s characteristics. Individualized PEEP titration guided by lung mechanics [52], pressure–volume curve [53], driving pressure [14], electrical impedance tomography [54] and others could result in different outcomes. Indeed, a recent individual patient data meta-analysis showed an association between high driving pressure and mortality in patients with ARDS [55]. Another individual patient data meta-analysis showed an association between high driving pressure and the occurrence of postoperative pulmonary complications in surgery patients receiving intraoperative ventilation [56]. So far there have been no reports on associations between driving pressure and outcome in patients without ARDS, but seen the two recent meta-analyses one could expect a similar association in these patients. It is important to realize that aiming for the lowest driving pressure is not similar to ventilation at high (or higher) PEEP levels. Indeed, in the meta-analysis of studies in patients with ARDS there were patients ventilated at high (or higher) PEEP levels while having a higher driving pressure than patients ventilated at high (or higher) PEEP levels [55]. A similar result came from the meta-analysis of studies in surgical patients, where a rise in PEEP could result in a lower but also a higher driving pressure, with associated better or worse outcomes [56]. These findings suggest that ventilation at higher PEEP levels could benefit one patient, where it recruits lung tissue resulting in a lower driving pressure, while harming another, where it causes overdistension resulting in a higher driving pressure [56, 57]. More studies are necessary to address the impact of the driving pressure in patients with and without lung injury, and how PEEP effects outcome through changes in the driving pressure.

The duration of ventilation was similar between patients ventilated with lower or higher levels of PEEP. However, one could consider it possible that physicians tend to extubate patients only at the ‘lowest’ PEEP level [58, 59] and to give more sedatives with the use of higher PEEP. Both could lengthen the weaning process. A recent post hoc analysis of two RCTs in surgical ICU patients showed that a change from using higher PEEP to lower PEEP was associated with a shorter duration of ventilation [60]. In fact, the use of higher levels of PEEP could lead to hypotension, and the use of high volumes of fluids to correct it could result in worse outcomes [50, 61].

It may be incorrect to assume that beneficial or harmful effects of PEEP are linear to its height. Like with many physiologic effects the effects of PEEP could be U-shaped [62–64], meaning that too low as well as too high levels of PEEP could be harmful and that the best level of PEEP is somewhere in between. Notably, the final shape of the curve could very well depend on severity of lung injury, and this could be one reason for why one individual patient data meta-analysis suggests higher levels of PEEP only to have beneficial effects in patients with more severe ARDS, and not in patients with less severe ARDS in whom higher levels of PEEP may have resulted more in overdistension than in resolution of atelectasis [12]. Also non-pulmonary effects of PEEP should be held in account, as high or higher levels of PEEP could reduce afterload of the left ventricle of the heart but at the same time decrease preload and increase afterload of the right ventricle of the heart. Furthermore, the effects of PEEP on the systemic circulation depend not only on how much lung tissue is recruited but also on lung volume, since if the lung volume is below the functional residual capacity at end expiration, an increase in the level of PEEP likely increases the cardiac output [14].

The results of this meta-analysis should be interpreted within the context of the included RCTs. Systematic reviews are subject to the overall quality of the studies and publication bias can occur. Additionally, we had a large variation of PEEP values and tidal volumes in the trials, so the type of ventilator setting was not always following strict protective ventilation strategies. Also, the levels considered as ‘higher PEEP’ in the included studies were different from what is called ‘higher PEEP’ nowadays. Furthermore, in our analysis of surgical ICU patients, the majority of the patients were ventilated after cardiac surgery and the duration of surgery was not accurately described. Also, lower PEEP was actually no PEEP, or ‘ZEEP’. However, a subgroup analysis including only RCTs using ZEEP in the lower PEEP arm found no differences compared to the overall analysis. The small sample size and the fact that the studies analyzed were from the era before use of low tidal volumes and mainly assessed outcomes that could suffer from bias like development of pulmonary complications were other limitations. The fact that practically all outcomes were only reported by some eligible trials is another limitation. Indeed, unreported outcomes could lead to overestimation of effects in meta-analyses [65]. The present meta-analysis did not have predefined levels of PEEP. Although it would have been interesting to look at the effects of different levels of PEEP, and not just ‘lower’ versus ‘higher’ or no PEEP versus any level of PEEP, this was not possible seen the available number of studies per each comparison. Ideally we would have performed an individual patient data meta-analysis, but seen the fact that some of the studies were very old, we did not try to get these data. Finally, the presence of moderate to high heterogeneity in several analyses decreases the strength of the findings.

## Conclusion

In conclusion, this systematic review and meta-analysis did not find a reduction in in-hospital mortality or a shorter duration of ventilation in patients ventilated with higher levels of PEEP. However, hypoxemia was less frequently seen with the use of higher PEEP and ARDS developed less frequently. The quality of the analyzed RCTs was low or very low. A well-powered high-quality RCT comparing higher versus lower levels of PEEP is very much needed.

## Additional file

**Additional file 1.** Online Supplement.

## Abbreviations

ARDS: acute respiratory distress syndrome
PEEP: positive end-expiratory pressure
ICU: intensive care unit
RCT: randomized controlled trials
RR: risk ratio
CI: confidence interval
SMD: standardized mean difference
ZEEP: zero end-expiratory pressure
SD: standard deviation
VILI: ventilation-induced lung injury
