# Supplementary material for: Associations between positive end-expiratory pressure and outcome of patients without ARDS at onset of ventilation: a systematic review and meta-analysis of randomized controlled trials
Source: Ann Intensive Care. 2016 Nov 3;6:109. doi: 10.1186/s13613-016-0208-7 (PMC5095097; doi:10.1186/s13613-016-0208-7)
Supplement: Supplementary file 1 — Additional file 1. Online Supplement. [file 13613_2016_208_MOESM1_ESM.docx]

**ASSOCIATIONS BETWEEN POSITIVE END-EXPIRATORY PRESSURE AND OUTCOME OF PATIENTS WITHOUT ARDS AT ONSET OF VENTILATION – a systematic review and meta-analysis of randomized controlled trials**

ONLINE SUPPLEMENT

Ary Serpa Neto MD MSc PhD, Roberto Rabello Filho MD, Thomas Cherpanath MD, Rogier Determann MD PhD, Dave A Dongelmans MD PhD,2,4 Frederique Paulus RN PhD, Pieter Roel Tuinman MD PhD, Paolo Pelosi MD FERS, Marcelo Gama de Abreu MD PhD, Marcus J Schultz MD PhD; for the PROVE Network Investigators

| **eTable 1 – Definition of outcomes in the included trials** | | | | | | |
| --- | --- | --- | --- | --- | --- | --- |
|  | **ARDS** | **Pneumonia** | **Atelectasis** | **Barotrauma** | **Hypoxemia** | **Hypotension** |
| **Surgical ICU Patients** | | | | | | |
| Michalopoulos *et al* | NA | NA | NA | Pneumothorax in chest X-ray | PaO_2_ / FiO_2_ < 100 | NA |
| Lago Borges *et al* | NA | NA | NA | NA | PaO_2_ / FiO_2_ < 300 | NA |
| Schmidt *et al* | PaO_2_ / FiO_2_ < 100 + RR > 36 mpm + alveolar infiltrates in chest X-ray | Not described | Not described | NA | NA | NA |
| Carroll *et al* | NA | NA | NA | Pneumothorax in chest X-ray | NA | MAP < 60 mmHg |
| Celebi *et al* | NA | NA | NA | Pneumothorax in chest X-ray | NA | NA |
| **Medical Patients** | | | | | | |
| Manzano *et al* | American European Consensus Conference | Presence of new, persistent (24 hrs), or progressive radiographic infiltrate on chest X-ray plus at least two of the following criteria: fever ≥ 38°C or hypothermia ≤ 35°C, leukocytosis ≥ 12 x 10^9^/L or leukopenia < 3 x 10^9^/L, or purulent tracheobronchial secretions | Presence of infiltrates or airless area in chest X-ray without signs of infection | Pneumothorax, pneumomediastinum or subcutaneous emphysema in chest X-ray | PaO_2_ / FiO_2_ < 175 causing modification in the amount of PEEP used | NA |
| Pepe *et al* | PaO_2_ / FiO_2_ < 150 + new diffuse radiogaphically infiltrates + PAWP < 18 | Change in infiltrate in chest X-ray + purulent tracheal aspirate + grown of know pathogens + fever + leukocytosis | Based on chest X-Ray | Pneumothorax, pneumomediastinum or subcutaneous emphysema in chest X-ray | NA | NA |
| Nelson *et al* | NA | NA | NA | Pneumothorax in chest X-ray | NA | NA |
| Lesur *et al* | NA | NA | NA | NA | NA | MAP < 65 mmHg for at least 10 minutes *or*  < 60 mmHg for at least 5 minutes *or* intervention was mandatory |
| Weigelt *et al* | Diffuse interstitial infiltrate on chest X-Ray + pulmonary venoarterial shunt > 25% + PaO_2_ < 250 | NA | NA | NA | NA | NA |
| *ARDS: acute respiratory distress syndrome; NA: not assessed; RR: respiratory rate/ MPM: movements per minute; MAP: mean arterial pressure; PEEP: positive end expiratory pressure; PAWP: pulmonary artery wedge pressure*  Only trials which reported these outcomes were included | | | | | | |

| **eTable 2 – Characteristics of included studies** | | | | | | | | | |
| --- | --- | --- | --- | --- | --- | --- | --- | --- | --- |
| **Study** | **Year** | **Type of Patients** | **N** | **High PEEP Group** | | | **Low PEEP Group** | | |
|  |  |  |  | **N** | **PEEP** | **Titration of PEEP** | **N** | **PEEP** | **Titration of PEEP** |
| **Surgical ICU Patients** | | | | | | | | | |
| Lago Borges *et al* | 2014 | Post-CG | 136^**^ | 44 | 10 | Arbitrarily | 45 | 05 | Arbitrarily |
| Lago Borges *et al* | 2013 | Post-CG | 136^**^ | 44 | 10 | Arbitrarily | 45 | 05 | Arbitrarily |
| Celebi *et al* | 2007 | Post-CG | 60^*^ | 20 | 10 | Decremental by PaO_2_ | 20 | 05 | Arbitrarily |
| Holland *et al* | 2007 | Post-CG | 28 | 14 | 10 | Arbitrarily | 14 | 05 | Arbitrarily |
| Dyhr *et al* | 2002 | Post-CG | 16 | 08 | 15 | 1 cmH_2_O above the LIP | 08 | 00 | Arbitrarily |
| Michalopoulos *et al* | 1996 | Post-CG | 67^b^ | 21 | 10 | Arbitrarily | 22 | 00 | Arbitrarily |
| Carroll *et al* | 1988 | Post-Surgery  P/F < 200 | 50 | 22 | 15 | Incremental by PaO_2_ or venoarterial admixture | 28 | 04 | Incremental by PaO_2_ / FiO_2_ |
| Marvel *et al* | 1986 | Post-CG | 44^a^ | 12 | 10 | Arbitrarily | 15 | 05 | Arbitrarily |
| Murphy *et al* | 1983 | Post-CG | 139 | NA | 10 | Arbitrarily | NA | 00 | Arbitrarily |
| Zurick *et al* | 1982 | Post-CG | 83 | 41 | 10 | Arbitrarily | 42 | 00 | Arbitrarily |
| Good *et al* | 1979 | Post-CG | 24 | 10 | 06 | Arbitrarily | 14 | 00 | Arbitrarily |
| Schmidt *et al* | 1976 | Post-Surgery  Risk for ARDS | 112 | 56 | 08 | Arbitrarily | 56 | 00 | Arbitrarily |
| **Medical** | | | | | | | | | |
| Ma *et al* | 2014 | NPE | 120 | 60 | 11-30 | Arbitrarily | 60 | 3-10 | Arbitrarily |
| Lesur *et al* | 2010 | ARF | 63 | 30 | 05 | Arbitrarily | 33 | 00 | Arbitrarily |
| Manzano *et al* | 2008 | Clinical  P/F > 250 | 127 | 64 | 5-8 | Arbitrarily | 63 | 00 | Arbitrarily |
| Vigil *et al* | 1996 | Trauma | 44 | 23 | 05 | Arbitrarily | 21 | 00 | Arbitrarily |
| Cujec *et al* | 1993 | ARF | 46 | NA | 10 | Arbitrarily | NA | 00 | Arbitrarily |
| Nelson *et al* | 1987 | P/F < 250 | 38 | 20 | 15 | Incremental until PaO_2_ / FiO_2_ > 300 or shunt < 0.2 | 18 | 08 | Arbitrarily |
| Pepe *et al* | 1984 | Risk for ARDS | 92 | 44 | 08 | Arbitrarily | 48 | 00 | Arbitrarily |
| Weigelt *et al* | 1979 | Risk for ARDS | 79 | 45 | 05 | Arbitrarily | 34 | 00 | Arbitrarily |
| Feeley *et al* | 1975 | ARF | 25 | 12 | 05 | Arbitrarily | 13 | 00 | Arbitrarily |
| *PEEP: positive end expiratory pressure (in cmH_2_O); NA: not available; LIP: lower inflection point* | | | | | | | | | |

| **eTable 3 – Summary characteristics of the included studies** | | |
| --- | --- | --- |
| Studies aiming at using V_T_ ≤ 8 ml/kg | 7 / 21 (33.3%) | |
| Studies in which low PEEP was 0 cm H_2_O PEEP | 13 / 21 (61.9%) | |
| Studies in which high PEEP was ≥ 5 cmH_2_O in | 5 / 21 (23.8%) | |
| Year of publication  < 20 years ago  ≥ 20 years ago | 8 / 21 (38.1%)  13 / 21 (61.9%) | |
|  | **High PEEP** | **Low PEEP** |
| Average number of patients | 30.3 | 31.5 |
| Tidal volume, ml/kg PBW | 9.8 ± 2.7 | 9.7 ± 2.8 |
| PEEP, cm H_2_O | 9.7 ± 4.0 | 2.0 ± 2.8 |
| Duration of ventilation, days | 3.4 | 2.7 |
| *V_T_: tidal volume; PEEP: positive end-expiratory pressure; ZEEP: zero PEEP* | | |

| **eTable 4 – Summary of findings table and quality of evidence using the GRADE approach** | | | | | | | |
| --- | --- | --- | --- | --- | --- | --- | --- |
| **Outcome** | **N of Studies**  **(participants)** | **Limitations** | **Inconsistency** | **Indirectness** | **Imprecision** | **Effect**  **(RR or SMD)** | **Quality of the Evidence** |
| Hospital Mortality | 7  (492) | Serious limitations | Serious inconsistency^**^ | No serious indirectness | Serious^b^ | 0.87 (0.62 – 1.21) | Low |
| 28-Day Mortality | 2  (183) | Serious limitations | Serious inconsistency^***^ | No serious indirectness | Serious^b^ | 0.55 (0.26 – 1.18) | Very low |
| Duration of Ventilation | 3  (125) | Serious limitations | Serious inconsistency^***^ | No serious indirectness | Serious^b^ | 0.68 (-0.24 – 1.61) | Very low |
| ARDS | 4  (410) | Serious limitations | Serious inconsistency^a^ | No serious indirectness | No Serious Imprecision | 0.43 (0.21 – 0.91) | Low |
| Pneumonia | 3  (331) | Serious limitations | Serious inconsistency^a^ | No serious indirectness | Serious^b^ | 0.58 (0.29 – 1.15) | Very low |
| Atelectasis | 3  (331) | Serious limitations | Serious inconsistency^a^ | No serious indirectness | Serious^b^ | 0.74 (0.33 – 1.66) | Very low |
| Barotrauma | 7  (534) | Serious limitations | Serious inconsistency^a^ | No serious indirectness | Serious^b^ | 1.20 (0.29 – 4.92) | Very low |
| PaO_2_ / FiO_2_ | 5  (380) | Serious limitations | Serious inconsistency^***^ | No serious indirectness | No Serious Imprecision | 0.72 (0.10 – 1.35) | Very low |
| Hypoxemia | 2  (170) | Serious limitations | Serious inconsistency^**^ | No serious indirectness | No Serious Imprecision | 0.42 (0.19 – 0.92) | Low |
| Blood Pressure | 2  (148) | Serious limitations | Serious inconsistency^a^ | No serious indirectness | Serious^b^ | -0.21 (-1.68 – 1.26) | Very low |
| Hypotension | 2  (141) | Serious limitations | Serious inconsistency^a^ | No serious indirectness | Serious^b^ | 5.27 (0.03 – 831.03) | Very low |
| *RR: risk ratio; SMD: standardized mean difference; ARDS: acute respiratory distress syndrome*  **: mild statistical heterogeneity  ***: high statistical heterogeneity  a: moderate statistical heterogeneity  b: large confidence interval | | | | | | | |

**eFigure 1 – Risk of Bias Graph**

**
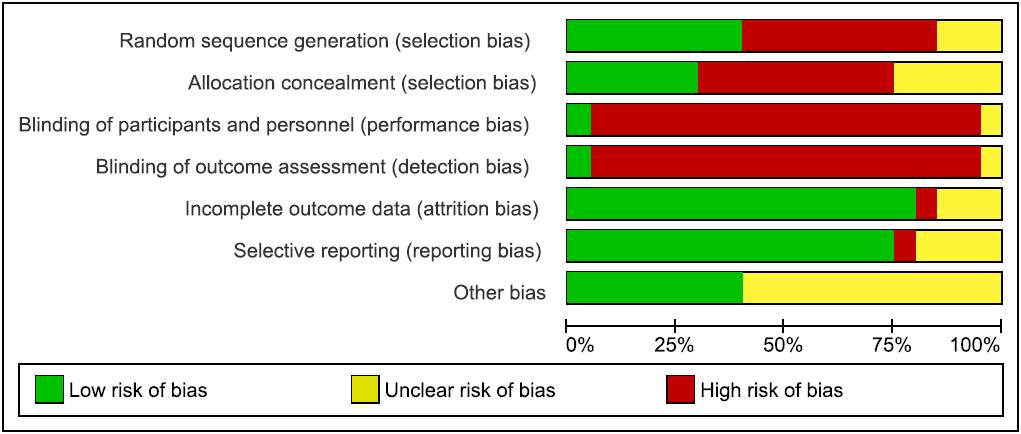
**

Risk of bias graph based on the Cochrane Risk of Bias Tool

**eFigure 2 – Risk of Bias Summary**

**
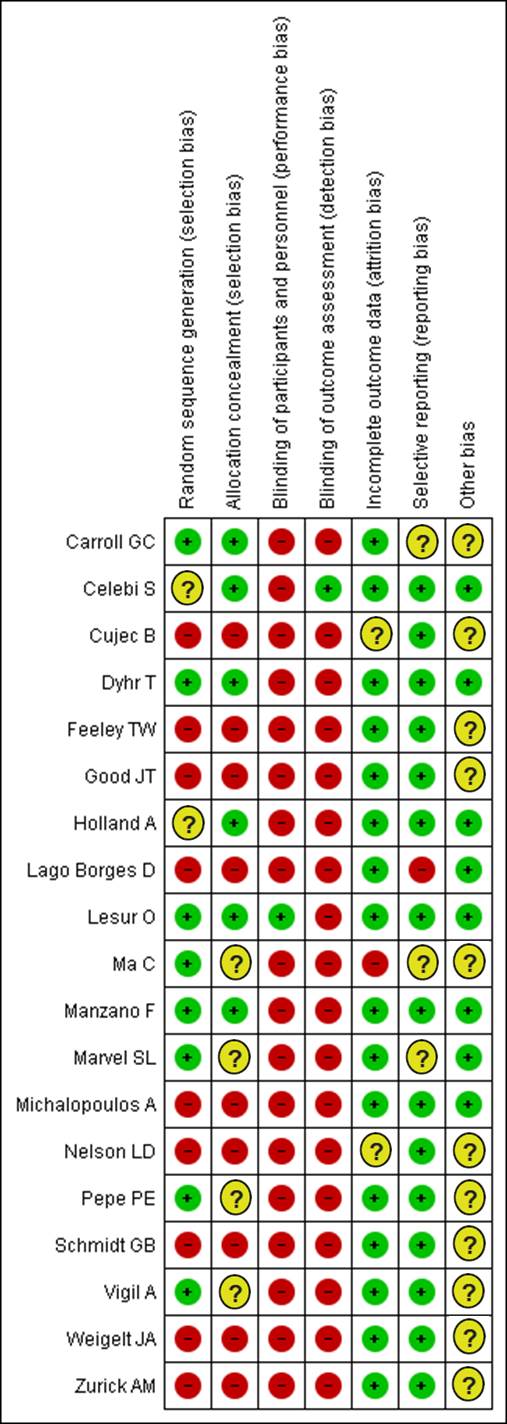
**

Risk of bias summary based on the Cochrane Risk of Bias Tool

**eFigure 3 – Meta-regression analysis for A) in-hospital mortality, B) ARDS, C) pneumonia, D) barotrauma, E) atelectasis and F) PaO_2_ / FiO_2_**

**
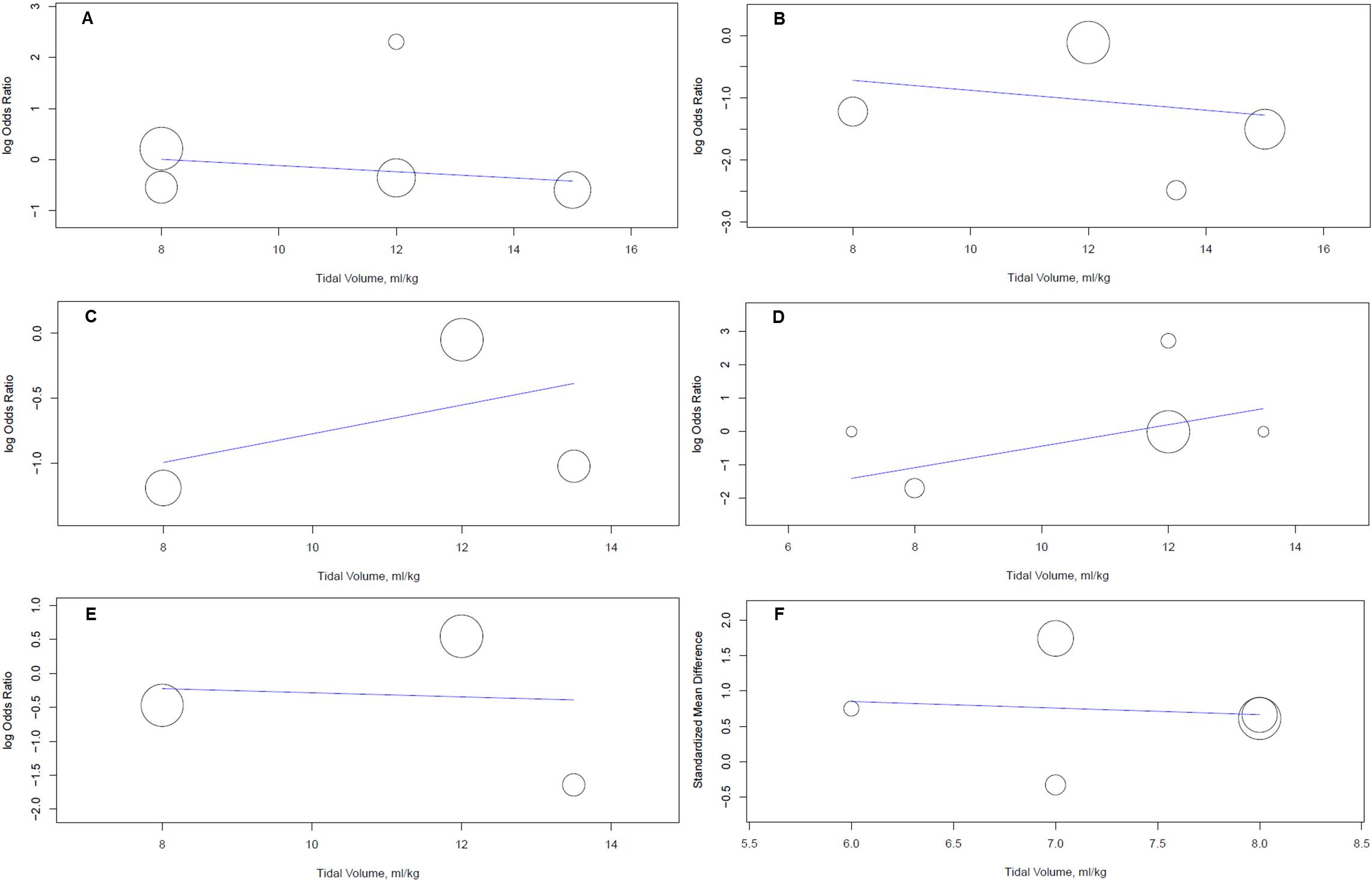
**

**eFigure 4 – Funnel plots**

**
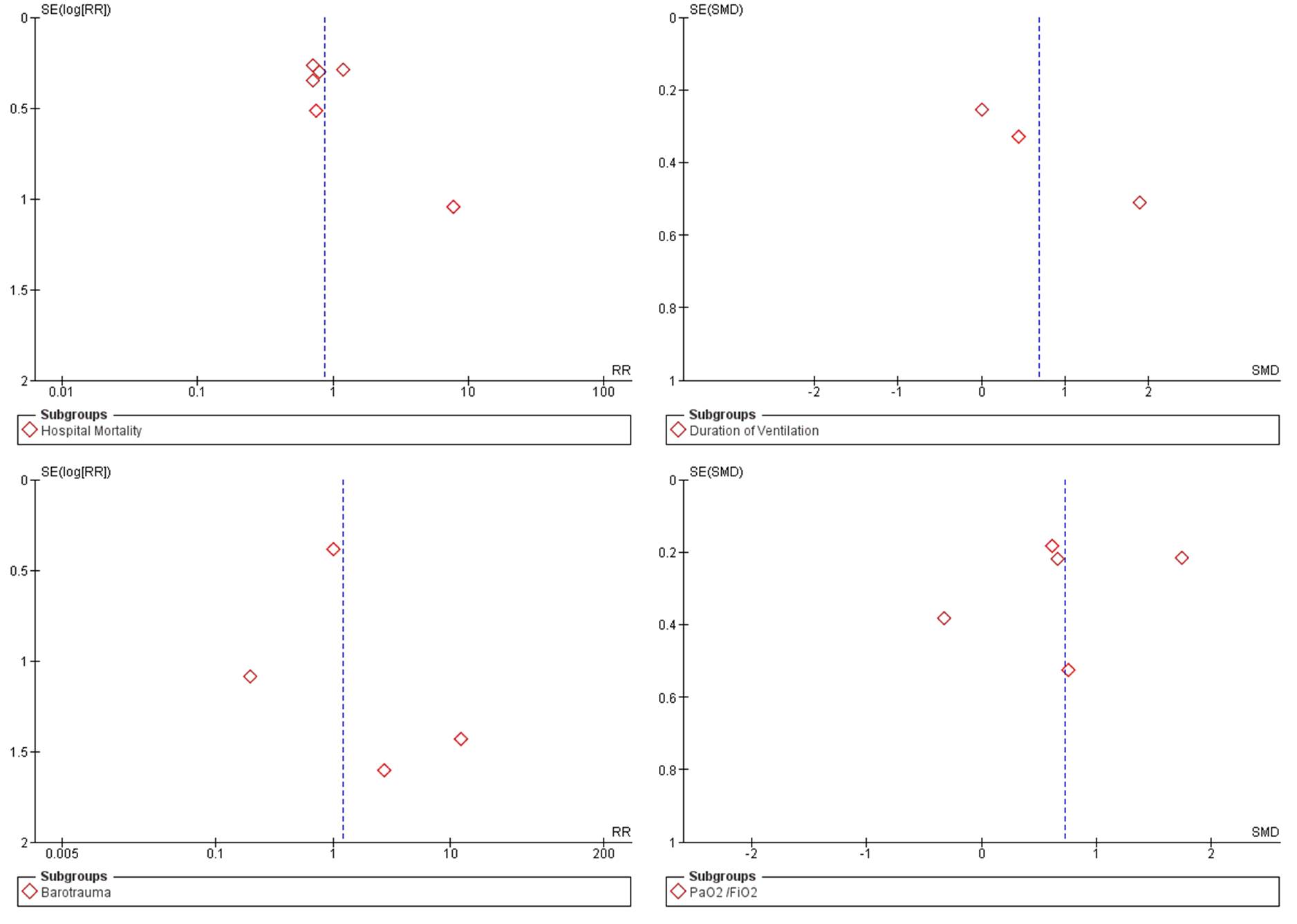
**

**eFigure 5 – Forest plot of clinical outcomes in medical patients. *High vs. Low PEEP compared***

**
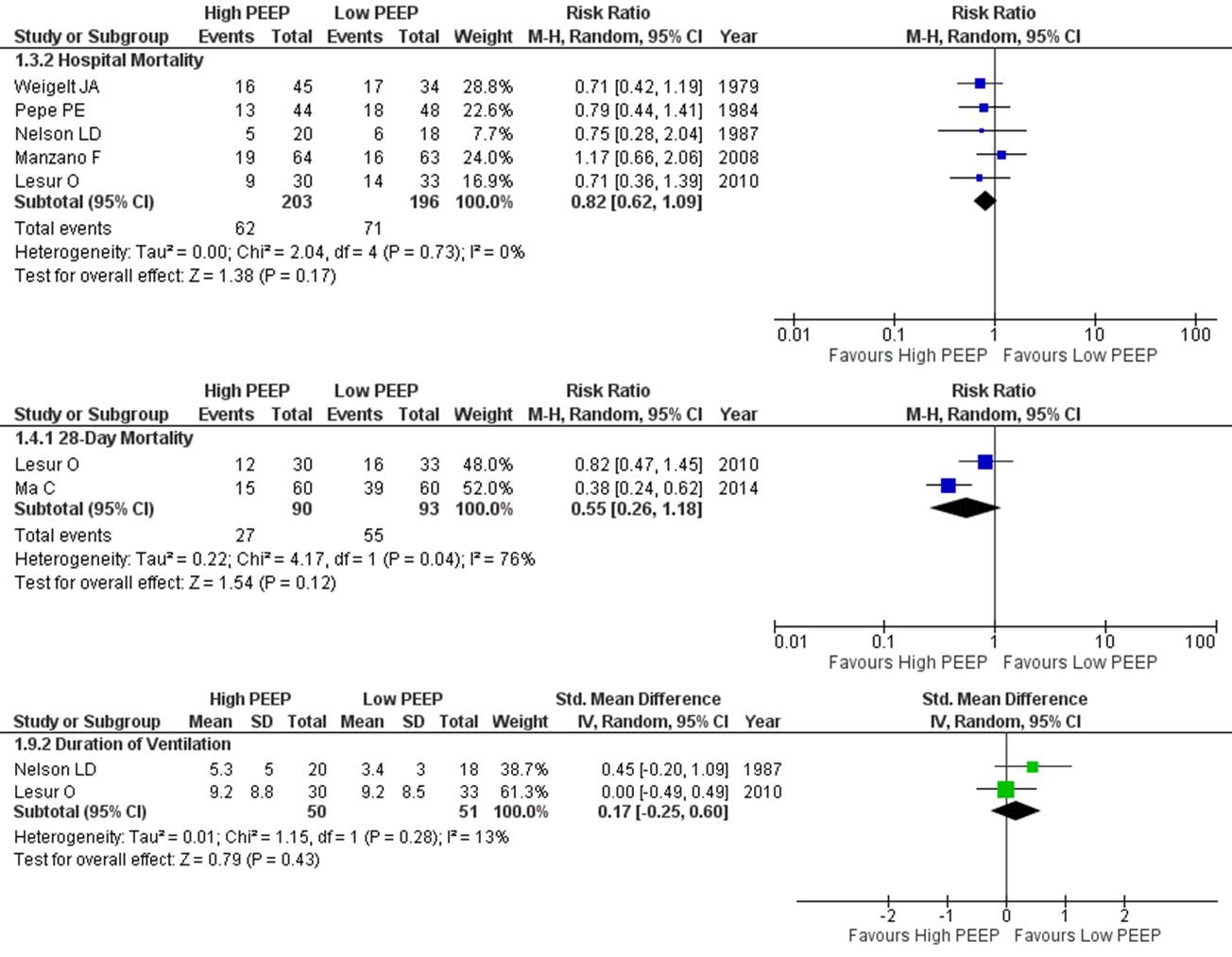
**

Forest plot of pulmonary complications in medical patients. *High vs. Low PEEP compared*

**eFigure 6 – Forest plot of pulmonary complications in medical patients. *High vs. Low PEEP compared***

**
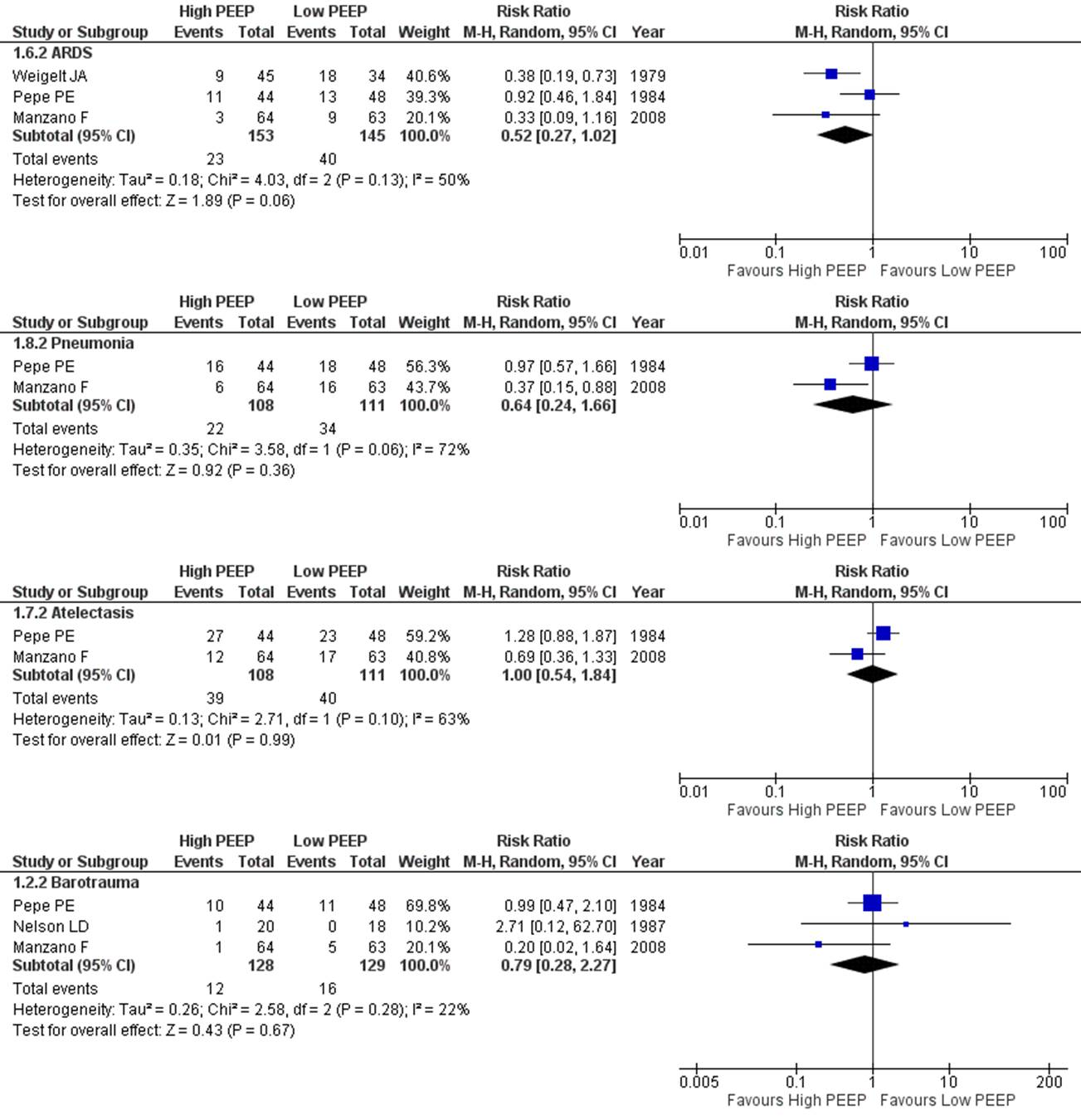
**

Forest plot of pulmonary complications in A) surgical ICU patients (left) and B) medical patients (right). *High vs Low PEEP*

**eFigure 7 – Forest plot of pulmonary and hemodynamic effects of PEEP medical patients. *High vs. Low PEEP compared***

**
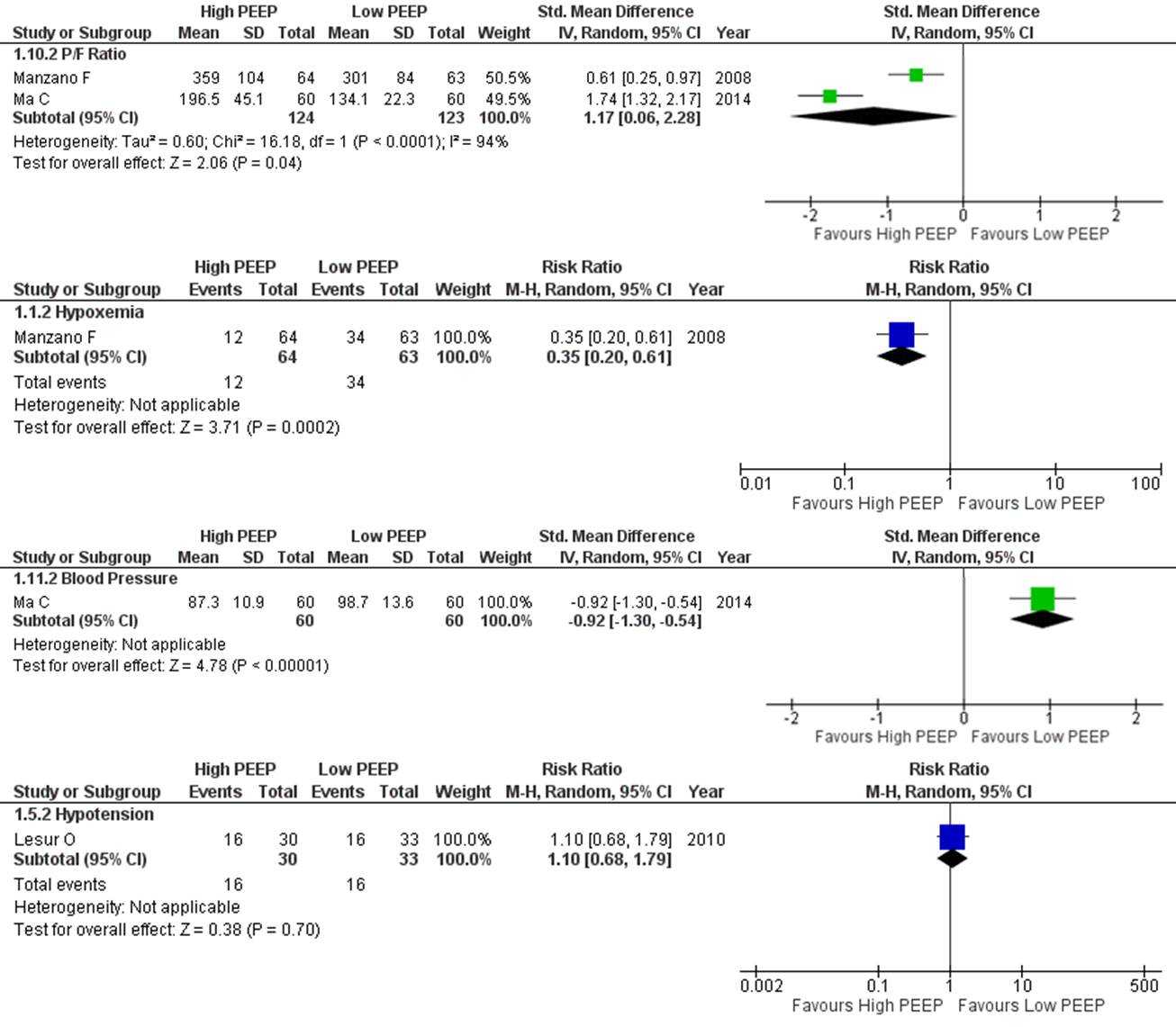
**

Forest plot of pulmonary and hemodynamic effects of PEEP in A) surgical ICU patients (left) and B) medical patients (right). *High vs Low PEEP*

**eFigure 8 – Forest plot of clinical outcomes comparing high PEEP to ZEEP**

**
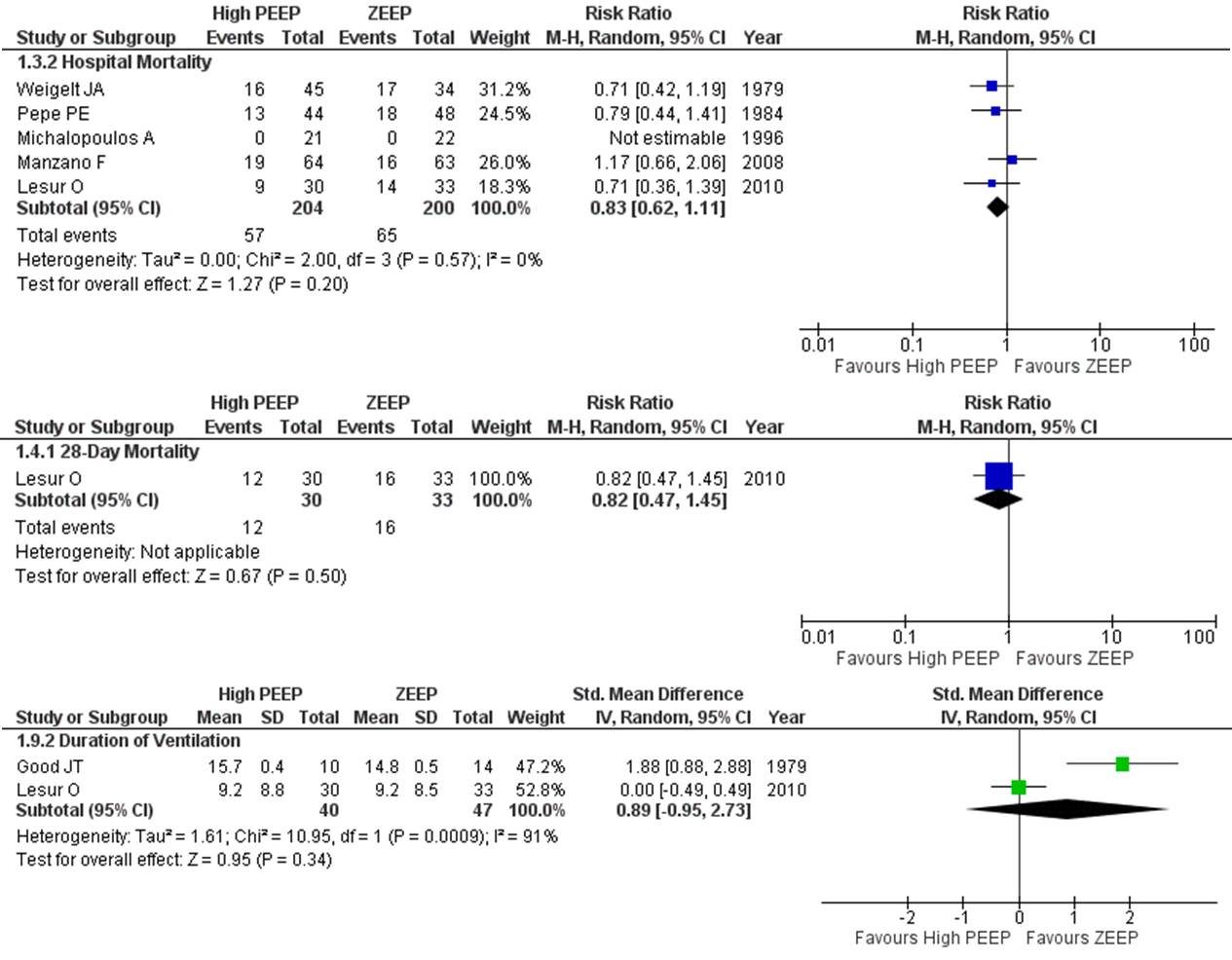
**

Forest plot of clinical outcomes comparing high PEEP to ZEEP

**eFigure 9 – Forest plot of pulmonary complications comparing high PEEP to ZEEP**

**
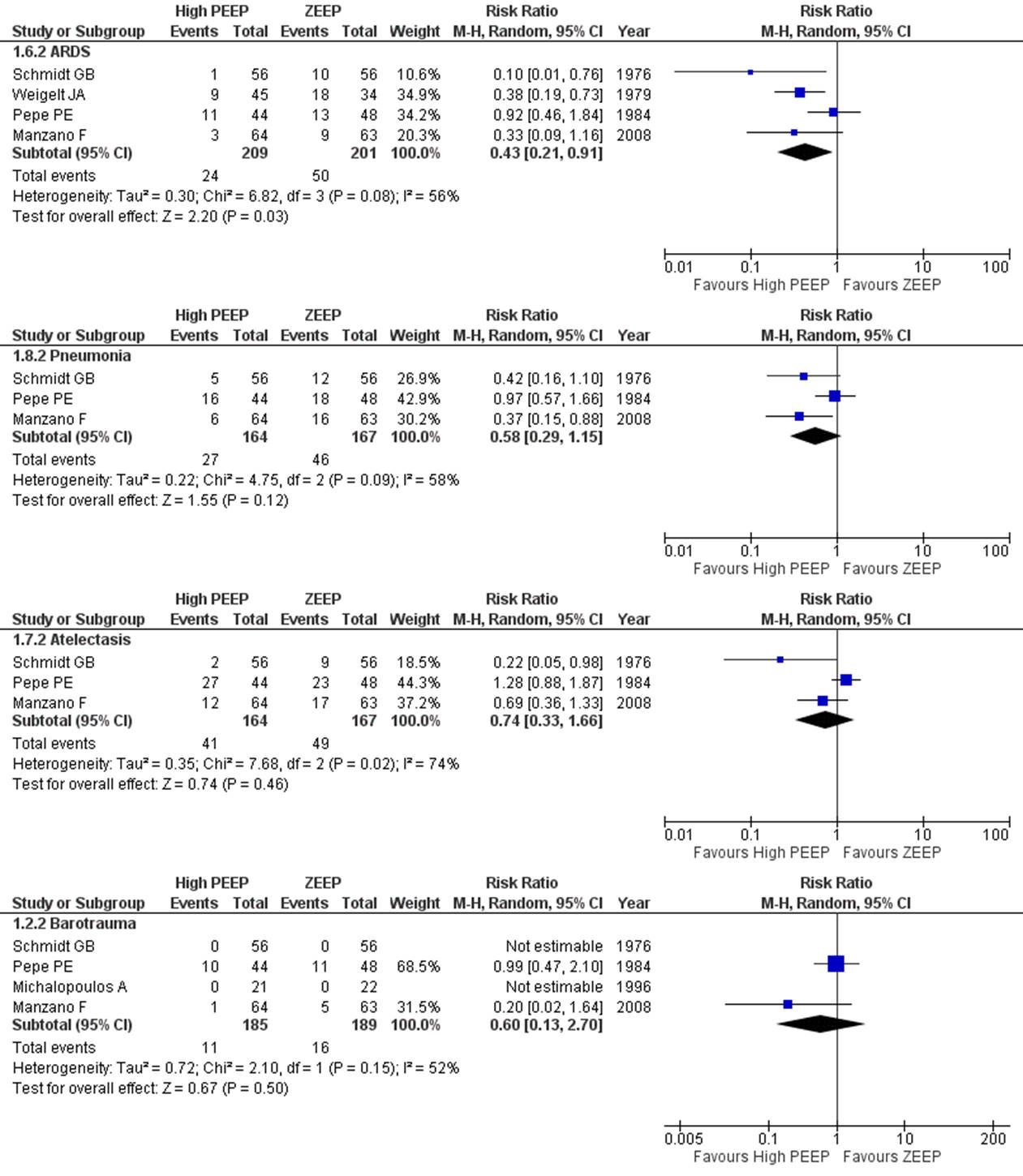
**

Forest plot of pulmonary complications comparing high PEEP to ZEEP

**eFigure 10 – Forest plot of systemic effects comparing high PEEP to ZEEP**

**
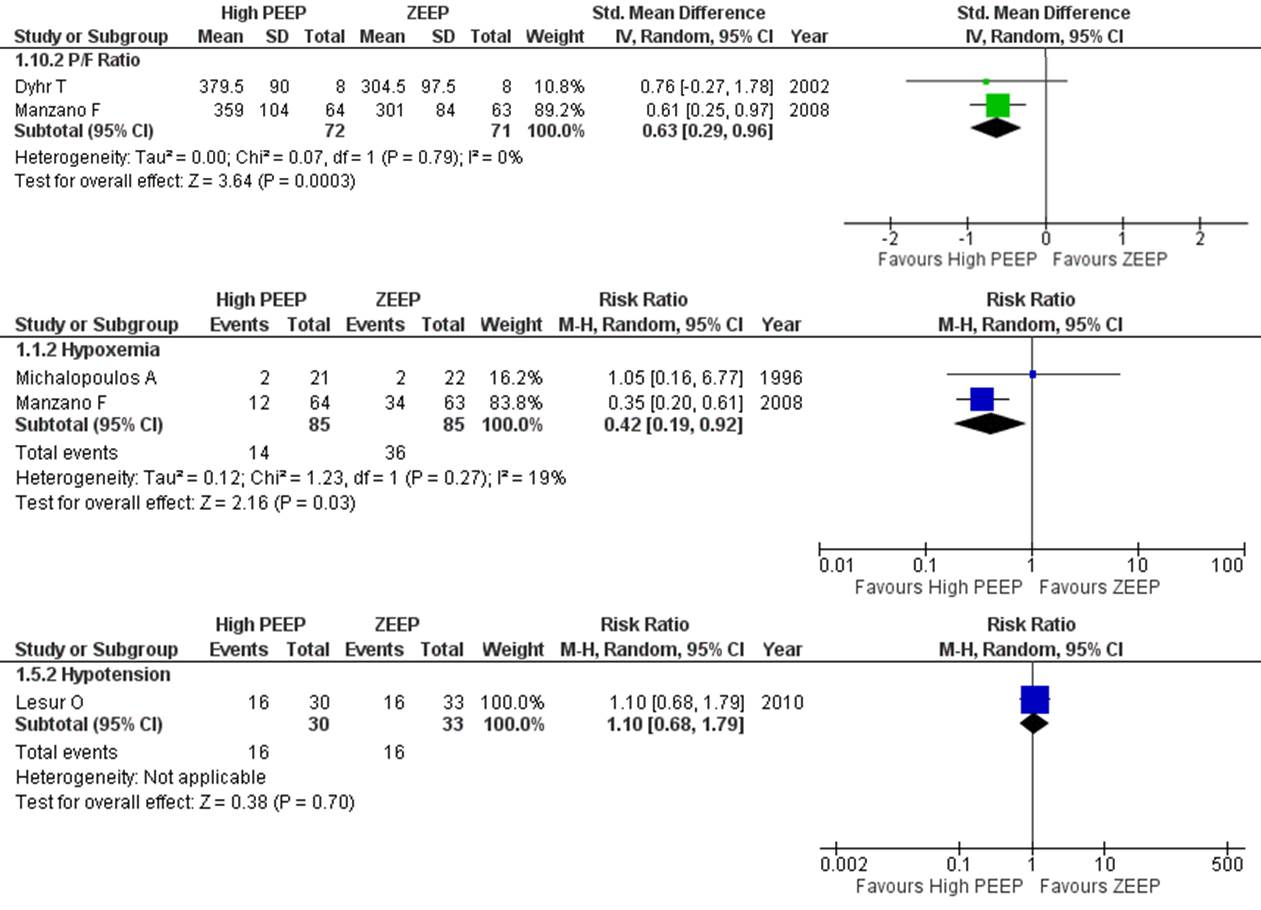
**

Forest plot of systemic effects comparing high PEEP to ZEEP

**eFigure 11 – Forest plot of hospital mortality and 28-day mortality according to year of publication**

**
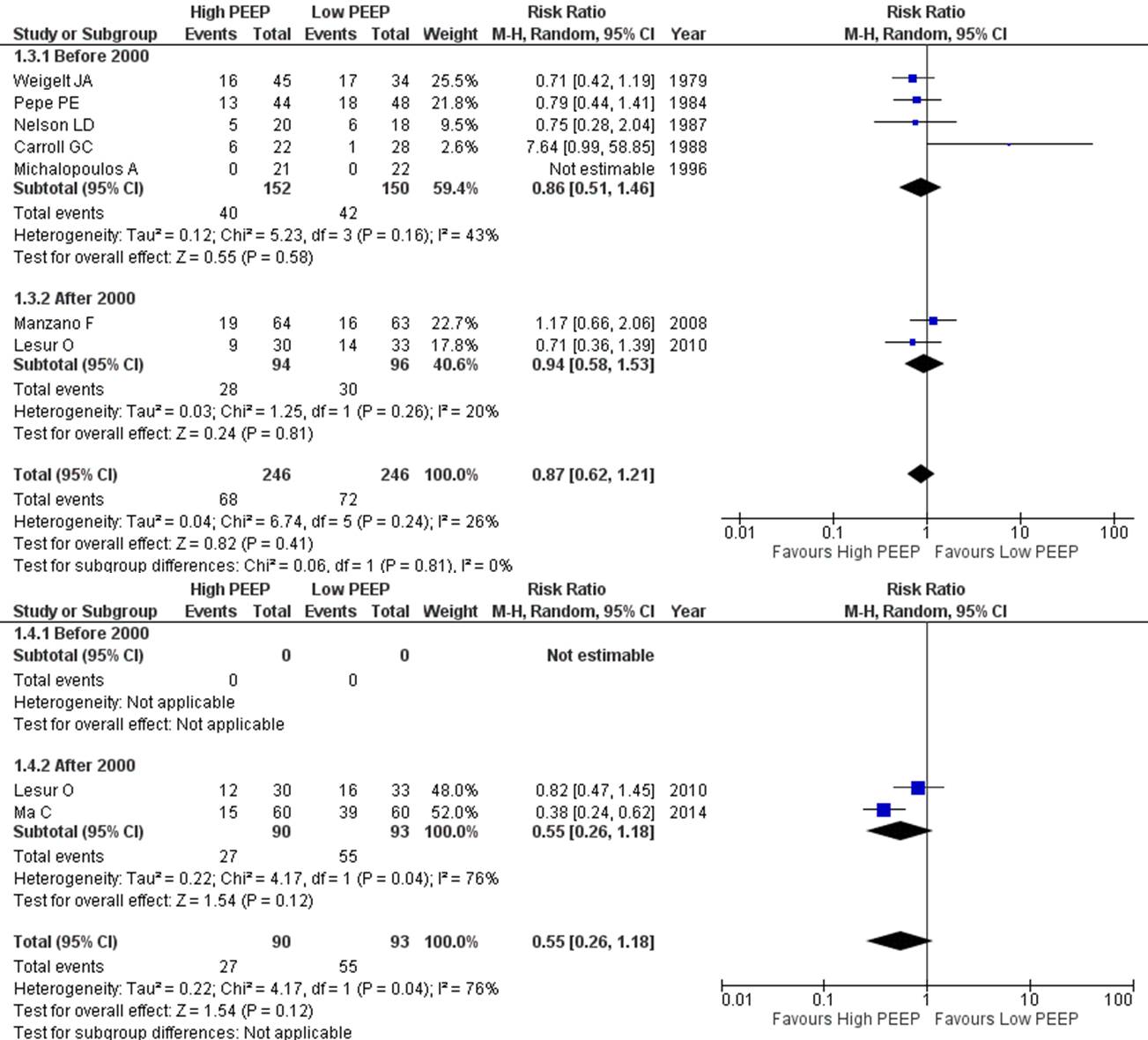
**

Forest plot of systemic effects comparing high PEEP to low PEEP according to year of publication

**eFigure 12 – Forest plot of ARDS, pneumonia, atelectasis and barotrauma according to year of publication**

**
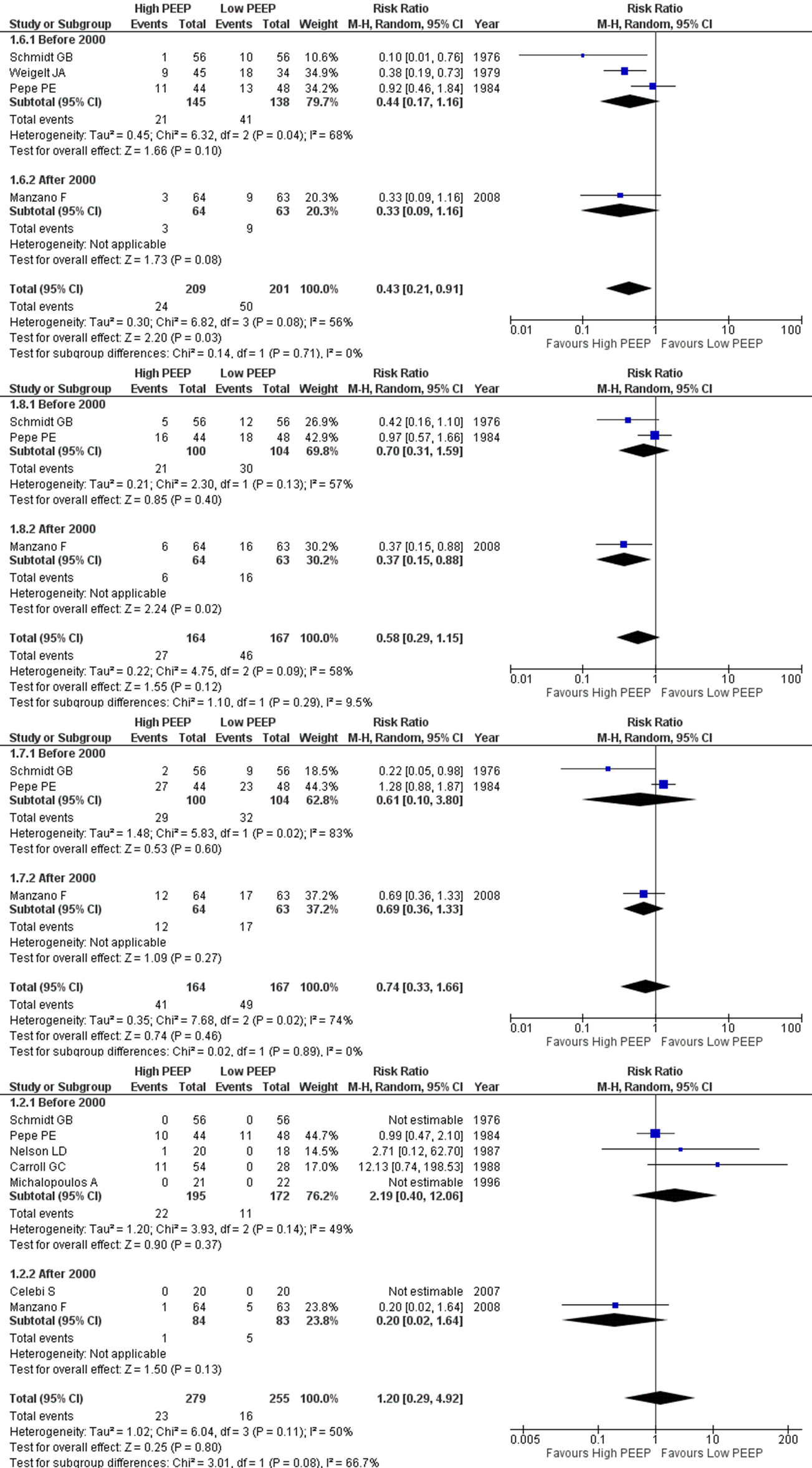
**

Forest plot of systemic effects comparing high PEEP to low PEEP according to year of publication

**eFigure 13 – Forest plot of hypoxemia and hypotension according to year of publication**

**
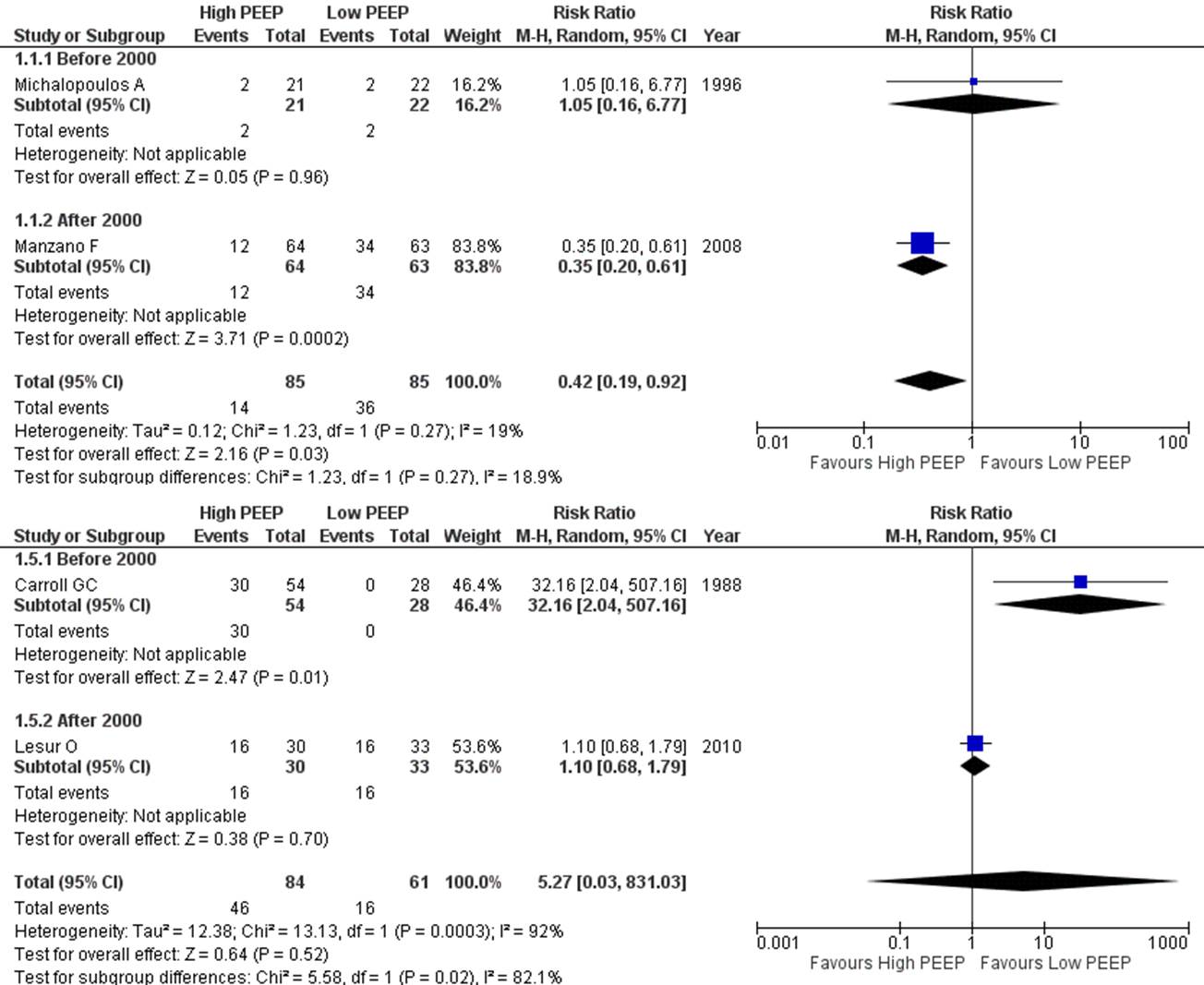
**

Forest plot of systemic effects comparing high PEEP to low PEEP according to year of publication
